# Elinzanetant in postmenopausal women with sleep disturbance: results from the randomized, phase II NIRVANA study

**DOI:** 10.1093/sleep/zsag208

**Published:** 2026-07-28

**Authors:** Fiona C Baker, Hadine Joffe, Claudio N Soares, Pauline M Maki, Malek Bajbouj, Rossella E Nappi, Gary Zammit, Cecilia Caetano, Ulrike Krahn, Huda Shalhoub, Frank Kramer, Hoi-Shen Radcliffe, Christian Seitz

**Affiliations:** Center for Health Sciences, SRI International, Menlo Park, CA, United States; School of Physiology, University of the Witwatersrand, Johannesburg, South Africa; Beth Israel Deaconess Medical Center, Harvard Medical School, Boston, MA, United States; Department of Psychiatry, Queen’s University, Kingston, ON, Canada; Department of Psychiatry, Psychology, and Obstetrics & Gynecology, University of Illinois Chicago, Chicago, IL, United States; Department of Psychiatry, Charité Universitätsmedizin Berlin, Berlin, Germany; German Center for Mental Health (DZPG), Berlin, Germany; Department of Clinical, Surgical, Diagnostic and Pediatric Sciences, University of Pavia, Pavia, Italy; Research Center for Reproductive Medicine, Gynecological Endocrinology and Menopause, IRCCS San Matteo Hospital, Pavia, Italy; Clinilabs Drug Development Corporation, Eatontown, NJ, United States; Bayer Consumer Care AG, Basel, Switzerland; Bayer AG, Wuppertal, Germany; Bayer AG, Berlin, Germany; Bayer AG, Wuppertal, Germany; Bayer PLC, Reading, United Kingdom; Bayer AG, Berlin, Germany; Institute of Clinical Pharmacology and Toxicology, Charité Universitätsmedizin Berlin, Berlin, Germany

**Keywords:** NIRVANA, menopause, sleep disturbances, elinzanetant, neurokinin-targeted therapy, neurokinin, vasomotor symptoms, Women’s health, clinical trials research, thermoregulation

## Abstract

**Study Objectives:**

Menopausal sleep disturbances can negatively impact quality of life. Elinzanetant, a dual neurokinin (NK)-1/NK-3 receptor antagonist, reduced self-reported sleep disturbance in postmenopausal women with moderate-to-severe vasomotor symptoms (VMS) in Phase III OASIS trials. NIRVANA explored elinzanetant’s effect on multimodal measures of objective and subjective sleep disturbance in postmenopausal women.

**Materials and Methods:**

Postmenopausal women (N = 110; 40–65 years) with moderate-to-severe VMS (≥20/week) and sleep disturbance (polysomnography [PSG] wakefulness after sleep onset [WASO] ≥30 min) were randomized 1:1 to receive elinzanetant 120 mg or placebo for 12 weeks. Sleep disturbance was assessed using PSG; daily Sleep Diary; and nightly contactless home-monitoring (Sleepiz One+ subgroup). The effect of elinzanetant versus placebo was analyzed using mixed-model repeated measures.

**Results:**

Elinzanetant was estimated to reduce PSG WASO at Week 4 versus placebo (least square [LS]-mean difference = –22.50 minutes, 90% CI: –35.47, –9.52). Minimal group differences between treatment arms were estimated at Week 12 for PSG WASO (LS-mean difference = –0.94 minutes, 90% CI: –13.96, 12.09). When including all available PSG data across the 12-week period, post hoc random coefficient modeling indicated treatment effects favoring elinzanetant for WASO, total sleep time, sleep efficiency, latency to persistent sleep, number of awakenings, arousal index, and % Stage N2 sleep. Treatment effects on WASO and awakenings favoring elinzanetant were observed with Sleepiz One+ and Sleep Diary across 12 weeks.

**Conclusion:**

In this sleep-focused study, elinzanetant reduced WASO at Week 4 and led to estimated improvements in sleep continuity over 12 weeks, supported by multimodal subjective and objective sleep outcomes.

## Graphical Abstract

**Graphical Abstract:**
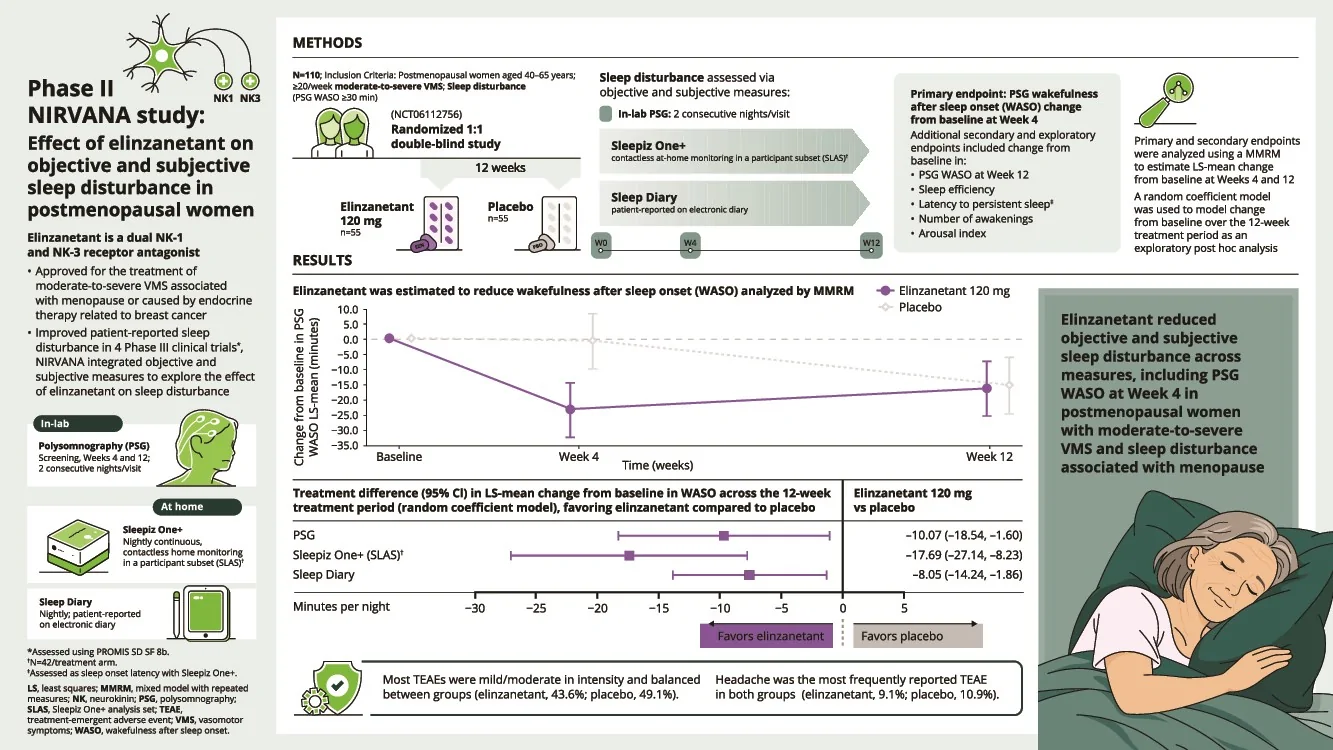

**Statement of Significance**

Menopausal sleep disturbances are common and disruptive, with a high unmet need due to their unique etiology and absence of specific treatments. NIRVANA is the first study to integrate gold-standard polysomnography; contactless, continuous home sleep monitoring; and patient-reported outcomes to assess treatment effects in postmenopausal women with vasomotor symptoms and sleep disturbance. Exploratory analyses from NIRVANA indicate consistent reductions in awakenings and improvements in sleep continuity across multimodal objective and subjective measures with elinzanetant, a dual neurokinin (NK)-1/NK-3 receptor antagonist. Together, these findings suggest that elinzanetant promotes a more stable sleep pattern, which appears to result in better subjective sleep quality, in postmenopausal women, a historically underserved group. Confirmatory studies are needed to corroborate this research.

## Introduction

The transitional period around menopause is associated with fluctuating, followed by declining, reproductive hormone levels. Physical and psychological symptoms associated with menopause can negatively impact quality of life and are correlated with long-term negative health outcomes [1]. Vasomotor symptoms (VMS, also known as hot flashes or night sweats if occurring during the night), characterized by a sensation of heat accompanying vasodilation, are reported by up to 80% of women during the menopausal transition and early postmenopause [2, 3]. Likewise, menopause-associated sleep disturbances are frequently observed during the menopausal transition and postmenopausal years, reported by up to 60% of women experiencing natural or surgical menopause [4, 5]. Common menopausal sleep disturbances, such as frequent nocturnal awakenings, are associated with functional impairment, poor quality of life, lost productivity, and increased healthcare use [6, 7]. Few studies have examined sleep architecture with polysomnography (PSG) at different stages of the menopausal transition and postmenopause and/or in relation to changes in reproductive hormones across menopause [8–12]. While some studies have found no differences in sleep architecture, others have shown better sleep, including more slow wave sleep (Stage N3), or only subtle differences in the sleep electroencephalogram. However, there is PSG-measured evidence that women with menopausal sleep complaints spent more time in wakefulness after sleep onset (WASO) and have a lower sleep efficiency compared with women in the menopausal transition without sleep complaints [13].

Menopause-associated sleep disturbances are unique in their underlying etiology, being at least partially driven by hormonal changes and often co-occurring with menopause-associated VMS and mood symptoms (i.e. depression, anxiety) that can further impact sleep [4, 14–18]. Aging-related physiological changes, such as modifications in circadian rhythm and decreased melatonin secretion, may also contribute to sleep difficulties during the menopausal transition [19]. Although VMS are strongly associated with poorer sleep quality and chronic insomnia, VMS-associated wake time accounts for less than 30% of wakefulness during the night in the menopausal transition, which suggests that other factors independent from VMS may also play a role in menopausal sleep disturbances [4, 8, 20–22]. Moreover, women without VMS show sleep disturbance in the menopausal transition, suggesting at least partially independent mechanisms underlying menopause-associated VMS and sleep disturbances [4, 23, 24]. Studies have shown that interventions to improve sleep, including cognitive behavioral therapy for insomnia and non-benzodiazepine hypnotics, are effective in menopausal women; however, they lack specificity for the distinct pathophysiological features of menopause-associated sleep disturbances [4, 25, 26]. Likewise, while menopausal hormone therapy can have a positive effect on perceived sleep quality among women with comorbid VMS, it is not considered a first-line approach for menopausal sleep disturbances, highlighting a key unmet need for novel treatment options targeting menopause-associated sleep disturbances [27].

Elinzanetant is a dual neurokinin (NK)-targeted therapy (NK-1 and NK-3 receptor antagonist) currently approved in multiple countries for the treatment of VMS associated with menopause and for the treatment of VMS due to endocrine therapy for breast cancer (in Europe) based on phase III trial data showing robust reductions in VMS frequency and severity [28–30]. NK-1 and NK-3 receptors and their respective ligands, substance P (SP) and neurokinin B (NKB), are expressed by hypothalamic kisspeptin/neurokinin B/dynorphin (KNDy) neurons, among others [31, 32]. During and after the menopausal transition, declining estradiol levels lead to hypertrophy and hyperactivation of KNDy neurons, disrupting thermoregulation and resulting in VMS [31, 33–35]. Along with a potential role in peripheral vasodilation, there is evidence that SP/NK-1 may have a role in primary insomnia as demonstrated by SP infusions leading to sleep disruption within a study population of healthy men [36, 37]. It is hypothesized that elinzanetant’s dual action as an NK-1/NK-3 receptor antagonist may lead to reduced menopause-associated sleep disturbance [38, 39].

Elinzanetant showed consistent and clinically meaningful reductions in patient-reported sleep disturbance in phase III randomized placebo-controlled trials for the treatment of moderate-to-severe VMS in postmenopausal women with natural or surgical menopause (OASIS-1, NCT05042362; OASIS-2, NCT05099159), including in a 52-week study (OASIS-3, NCT05030584) and in a separate population of women receiving endocrine therapy for hormone receptor-positive breast cancer (OASIS-4, NCT05587296) [28–30]. Consistent reductions in subjective sleep disturbance with elinzanetant in the OASIS trials inspired the sleep-focused NIRVANA trial (NCT06112756; https://clinicaltrials.gov/study/NCT06112756) [40], which aimed to develop a deeper understanding of elinzanetant’s impact on objective patterns of sleep and sleep disturbance. The primary objective of NIRVANA was thus to estimate the effect of elinzanetant on menopause-associated sleep disturbance compared to placebo in postmenopausal women experiencing moderate-to-severe VMS and sleep disturbance using in-lab PSG, the gold-standard technique for objective assessment of sleep parameters [41]. While PSG provides comprehensive, high-quality sleep data, the unfamiliar sleep environment and narrow observation window of one or two nights in a row mean that the in-lab snapshot may not fully capture an individual’s typical sleep pattern [42, 43]. Therefore, continuous at-home monitoring using the contactless Sleepiz One+ device was also performed as an exploratory objective to provide complementary insights. Together with patient reported outcomes (PROs) collected for consistency and comparison with the OASIS trials, these objective assessments provide a holistic view of sleep patterns and enable estimation of elinzanetant’s impact on both subjectively and objectively measured sleep parameters over time.

## Materials and methods

### Study design

NIRVANA (NCT06112756) was a phase II, multicenter, multi-country, double-blind, randomized, parallel-group, placebo-controlled, exploratory study to estimate the effect and assess safety of elinzanetant for the treatment of menopause-associated sleep disturbance. Furthermore, the NIRVANA study served to inform future trial design investigating sleep disturbance associated with menopause. The study included a 12-week intervention period, followed by a 4-week safety follow-up period, and no interim analysis was planned. The patient voice was captured via qualitative interviews prior to study initiation to inform the development of the endpoint measures, and specifically the choice of clinical outcome assessments and the concepts in line with FDA Patient Focused Drug Development Guidance Series (PFDD) [44–46].

NIRVANA enrolled postmenopausal women aged 40–65 years experiencing moderate-to-severe VMS (≥20/week) and sleep disturbance. Sleep disturbance was defined as WASO ≥30 minutes/night, computed as the mean of two screening PSG assessments, with neither night showing <20 minutes of WASO. Postmenopause was defined as serum follicle-stimulating hormone levels >40 mIU/mL and a serum estradiol concentration of <30 pg/mL at screening; women with either natural or surgical menopause were eligible. Moderate-to-severe VMS were defined as a “sensation of heat with sweating” that did (severe) or did not (moderate) cause cessation of activities, measured using a Hot Flash Daily Diary. Exclusion criteria included a medical history of, or baseline PSG assessment indicating, a sleep disorder diagnosis other than sleep disturbance associated with menopause (e.g. obstructive sleep apnea). The complete list of inclusion and exclusion criteria are provided in Table 1.

The sample size was determined based on precision considerations (i.e. the half width of the confidence interval) for estimating the treatment effect of elinzanetant compared to placebo in the change from baseline in WASO to Week 4. Assuming the pooled standard deviation is 33.2 minutes for the change from baseline in WASO at Week 4 [47], a total sample size of 70 participants (35 per arm) would give a 90% probability that the 2-sided 90% confidence interval will have a half width of 14.7 minutes or less. To account for missing data and possible inflation in variability due to missing data imputation, a total of approximately 78 participants was planned to be randomized. However, accelerating recruitment and a lower screening failure rate towards the end of the screening period resulted in a final sample size of 110. Participants were randomized using computer-generated random numbers at 1:1 to receive 120 mg (2 × 60 mg soft gel capsules) of elinzanetant or matching placebo orally once daily at night for 12 weeks. Randomization was stratified by average weekly frequency of moderate-to-severe VMS (<35, ≥35–<50, ≥50) as reported in daily diaries over the 14 days of screening. As this was a double-blind study, both participants and investigators were blinded to study intervention. Participants in the placebo group received soft gel capsules that were identical in size and appearance to the investigational product. Investigators remained blind to each participant’s assigned study intervention throughout the course of the study. In addition, the centralized reading of PSG data was blinded to study intervention. Efficacy and safety endpoints are described below.

### Efficacy assessments

#### Polysomnography

The primary objective of NIRVANA was to estimate the effect of elinzanetant on menopause-associated sleep disturbance as determined by PSG. In-lab PSG data (6 nights total) were collected during three visits of two consecutive nights each at screening, Week 4, and Week 12. Lights-off time was approximately the same bedtime self-reported by participants during screening and remained consistent across PSG study nights (+/− 30 minutes); lights-on time was 8 hours following lights-off. Participants who awoke naturally before the designated lights-on time were strongly encouraged to remain hooked up in bed for the full 8 hours. PSG recordings were scored centrally according to AASM Scoring Manual Version 3.0 guidelines [48].

PSG parameters reported here include total sleep time, sleep efficiency (total sleep time/time in bed ^*^ 100), and latency to persistent sleep, along with measures of sleep continuity and sleep architecture: WASO, number of awakenings, arousal index, and proportion/duration of time spent in Stage N1, N2, N3 and REM sleep. Parameters are defined in Table 2.

Change from baseline in WASO at Week 4 was the primary endpoint. Change from baseline in WASO at Week 12 was a secondary endpoint, as were changes from baseline in sleep efficiency at Weeks 4 and 12. Changes over time in all other PSG parameters were pre-specified exploratory endpoints.

#### Sleepiz One+

To complement in-lab PSG assessment, participants were invited to use the Sleepiz One+ (Sleepiz AG, Zurich, Switzerland) contactless home-monitoring device for nightly at-home sleep monitoring. The Sleepiz One+ device uses radar principle based on radiofrequency scanning and reflection (in the mm-wavelength range) to collect physiologic data during sleep in a contactless manner. It has shown good performance for detecting respiratory events during sleep [49], and preliminary evidence from an epoch-level comparison shows good agreement between Sleepiz One+ and PSG in postmenopausal women [50].

Use of the Sleepiz One+ device was voluntary, with separate informed consent. Data were collected nightly at home and during in-lab PSG assessment from screening through end of treatment. A third-party vendor hosted and transmitted the Sleepiz One+ data via their system. Effect of elinzanetant on sleep disturbance determined by Sleepiz One+ was an exploratory endpoint.

Sleepiz One+ parameters for analysis included total sleep time, sleep efficiency, sleep onset latency, WASO, and number of awakenings, as defined in Table 2. Of note, definitions for the same parameters varied slightly between objective assessment methods, in part because there were no markers for “lights off” and “lights on” with the Sleepiz One+ device. For example, Sleepiz One+ WASO was defined as “time awake between sleep onset (i.e. first sleep period >10 min) and the last awakening” whereas PSG WASO was defined as “time spent awake from onset of persistent sleep [i.e. 20 consecutive epochs [10 min] of non-wakefulness] to lights on”.

### Electronic patient-reported outcomes

Participants’ assessments of sleep disturbance were recorded using the PROMIS SD SF 8b and a sponsor-developed daily Sleep Diary, among others. All PRO data were collected at home and during study visits, captured via participant data entry using an electronic handheld device, which was provided, and transmitted to the vendor’s database system. Compliance was closely monitored by study center personnel and supported by automated reminders. The additional PROs (e.g. Insomnia Severity Index, Generalized Anxiety Disorder-7) assessed will be reported in another manuscript.

#### PROMIS SD SF 8b

Participants completed the PROMIS SD SF 8b questionnaire at screening and weekly from baseline until end of the 12-week treatment. The PROMIS SD SF 8b assesses the degree of sleep disturbance over the previous 7 days, with higher scores indicating greater severity. Raw scores were converted into T-scores for comparison with population norms and characterization of sleep disturbance: mild (55–60), moderate (60–70), severe (>70) [51]. Changes from baseline in PROMIS SD SF 8b total T score at Weeks 4 and 12 were secondary endpoints.

#### Sleep diary

Participants completed the Sleep Diary daily, in the morning after waking up, from screening until end of the 12-week treatment. Sleep Diary items captured time going to bed, time taken to fall asleep, number and duration of awakenings during the night, time waking up in the morning, and time getting out of bed. Sleep Diary parameters for analysis were derived from this information and included self-reported total sleep time, self-reported sleep efficiency, self-reported latency to persistent sleep, self-reported WASO, and self-reported number of awakenings, as defined in Table 2. Change from baseline in Sleep Diary parameters was an exploratory endpoint.

### Safety

Safety, a secondary objective of the NIRVANA study, was assessed throughout the treatment and follow-up periods via continuous monitoring of treatment-emergent adverse events (TEAEs), serious TEAEs, and TEAEs of special interest. The latter comprised postmenopausal uterine bleeding and potential liver events, i.e. any condition triggering close liver observation (e.g. alanine transaminase [ALT] and/or aspartate aminotransferase [AST] >8× upper limit of normal [ULN]; ALT and/or AST >3× ULN with total bilirubin >2× ULN). Physical exams, including vital signs, and clinical safety laboratory tests (hematology, clinical chemistry, coagulation, hormones, and/or urine analysis) were conducted at screening, Week 4, and Week 12. Adverse events were documented by site investigators in the electronic case report form. Blood and urine samples were analyzed at a central laboratory.

### Statistical analysis

No statistical hypothesis testing was planned or performed during the analysis since the primary objective of this study was to estimate the treatment effect of elinzanetant on sleep disturbance associated with menopause.

Endpoints and assessments related to efficacy and safety were analyzed in the full analysis set (FAS) and safety analysis set (SAF), respectively. The FAS included all participants randomly assigned to study treatment, with participants analyzed as per their randomized study treatment according to intention to treat principles. The SAF included all participants randomly assigned to study treatment who were exposed to study treatment at least once; participants in the SAF were analyzed according to study treatment received. The Sleepiz One+ analysis set (SLAS), a subset of participants with at least one Sleepiz One+ evaluation during the study period, was used to analyze sleep endpoints assessed via the Sleepiz One+ device. Results of pre-specified secondary and exploratory endpoints not discussed in this manuscript will be reported elsewhere.

#### Analysis of PSG-derived parameters

For the pre-specified main analysis (primary and supplementary estimand) of PSG-derived WASO, the mean value of the two consecutive nights at each visit was calculated. If a participant only had one PSG night for a given visit, the data from that one night were used for the analysis. The primary and secondary endpoints (i.e. changes from baseline to Weeks 4 and 12 in WASO) were analyzed using a mixed model with repeated measures (MMRM). The model included baseline value, study treatment, average weekly frequency of moderate-to-severe VMS (stratification factor: <35, ≥35–<50, ≥50), and week as covariates, plus a treatment-by-week interaction term. An unstructured covariance structure was used. The least squares mean (LS-mean) change from baseline and standard error (SE) for each treatment group, plus the LS-means difference (elinzanetant vs placebo) and corresponding 90% confidence interval (CI), were estimated from the model at Weeks 4 and 12.

A supplementary estimand of WASO using log-transformed data was pre-specified to address possible deviations from the normal distribution. The least squares geometric mean (LSGM) ratio to baseline and SE for each treatment group, plus the LSGM treatment ratio (elinzanetant vs placebo) and corresponding 90% CI, were estimated from the model at Weeks 4 and 12.

Prior to performing the MMRM model for WASO, missing data were handled using a hypothetical strategy for each of the three pre-specified intercurrent events (ICEs): premature discontinuation of study treatment, temporary study treatment interruption, and intake of prohibited concomitant medication having an impact on efficacy. This strategy estimated the full efficacy potential of elinzanetant in the ideal situation if participants adhered to randomized study treatment without taking prohibited concomitant medications impacting sleep. Any data that occurred after premature discontinuation of study treatment were discarded. For temporary treatment interruption and intake of prohibited concomitant medication, only data during the timeframe of the ICE were discarded.

Inter-night variability, including between consecutive nights of PSG assessment at each visit, was noted in both treatment groups. This observation prompted interest in also assessing the overall effect over the entire study period rather than at a single timepoint and underpinned a broader evolution in analytic approach to consider the data holistically. Therefore, a random coefficient model including all available timepoints (i.e. each overnight PSG recording considered separately) was used to model change from baseline over the 12-week treatment period as an exploratory post hoc analysis. Change from baseline at individual timepoints was the dependent variable. Fixed effects were the same as in the main analysis, except that week and treatment-by-week interaction were replaced by study day and study treatment-by-study day interaction. For other PSG-derived parameters, similar random coefficient models were applied.

Estimated LS-mean change from baseline for each treatment group, plus the LS-means difference (elinzanetant vs placebo), are presented, as are the respective statistical measures based on log-transformed data (i.e. LSGM ratios).

#### Analysis of Sleepiz One+ parameters

For Sleepiz One+ data, a random coefficient model was used to model change from baseline over the 12-week treatment period as an exploratory post hoc analysis, as described above. Baseline was derived using all available data assessed at home with a good signal duration (GSD) of 4 or more hours prior to starting the treatment. GSD is a qualifier used to evaluate data quality over a period of time and participant compliance using the device. It measures the daily total duration where the participant is in sight of the device and where either movement, breathing rate, or heart rate can be computed.

The random coefficient model used data collected across the 12-week treatment period, as measured by Sleepiz One+ at home on nights with GSD ≥4 hours; it did not include data collected during in-lab PSG assessment, which is not representative of routine home use.

#### Analysis of PRO data

Random coefficient models including all available timepoints for PROMIS SD SF 8b and daily ratings of subjective sleep parameters from the Sleep Diary were used to model change from baseline over the 12-week treatment period as an exploratory post hoc analysis, as described above.

### Ethical and regulatory considerations

The study was conducted in accordance with the Declaration of Helsinki, the Council for International Organizations of Medical Sciences (CIOMS) International Ethical Guidelines, applicable International Council for Harmonization of Technical Requirements for Pharmaceuticals for Human Use Good Clinical Practice (ICH GCP) guidelines, and applicable laws and regulations. It was reported using the Consolidated Standards of Reporting Trials (CONSORT) guidelines. Institutional review board and ethics committee approval were obtained for all study sites. All participants provided written informed consent.

## Results

### Sample characteristics

The study was conducted at 39 centers between November 2023 and November 2024; 30 of these sites (13 in Europe, 17 in the United States) randomized subjects. Separate sleep laboratories were used by 9 US sites that did not have on-site PSG capability. A flow diagram depicting participant disposition is summarized in Figure 1. Of the 338 women screened, 110 were randomized and received treatment (FAS/SAF: 55 elinzanetant, 55 placebo), and 103 completed the treatment phase (52 elinzanetant, 51 placebo). For those who did not proceed to randomization, screening failure (223/228, 97.8%) was the primary reason. Three (5.5%) participants in the elinzanetant group and four (7.3%) participants in the placebo group discontinued the study during the treatment phase, most commonly due to AEs. Occurrence of ICEs was low; treatment interruption was the most common, reported for 6 (10.9%) participants in the elinzanetant group and 9 (16.4%) participants in the placebo group.

**Figure 1:**
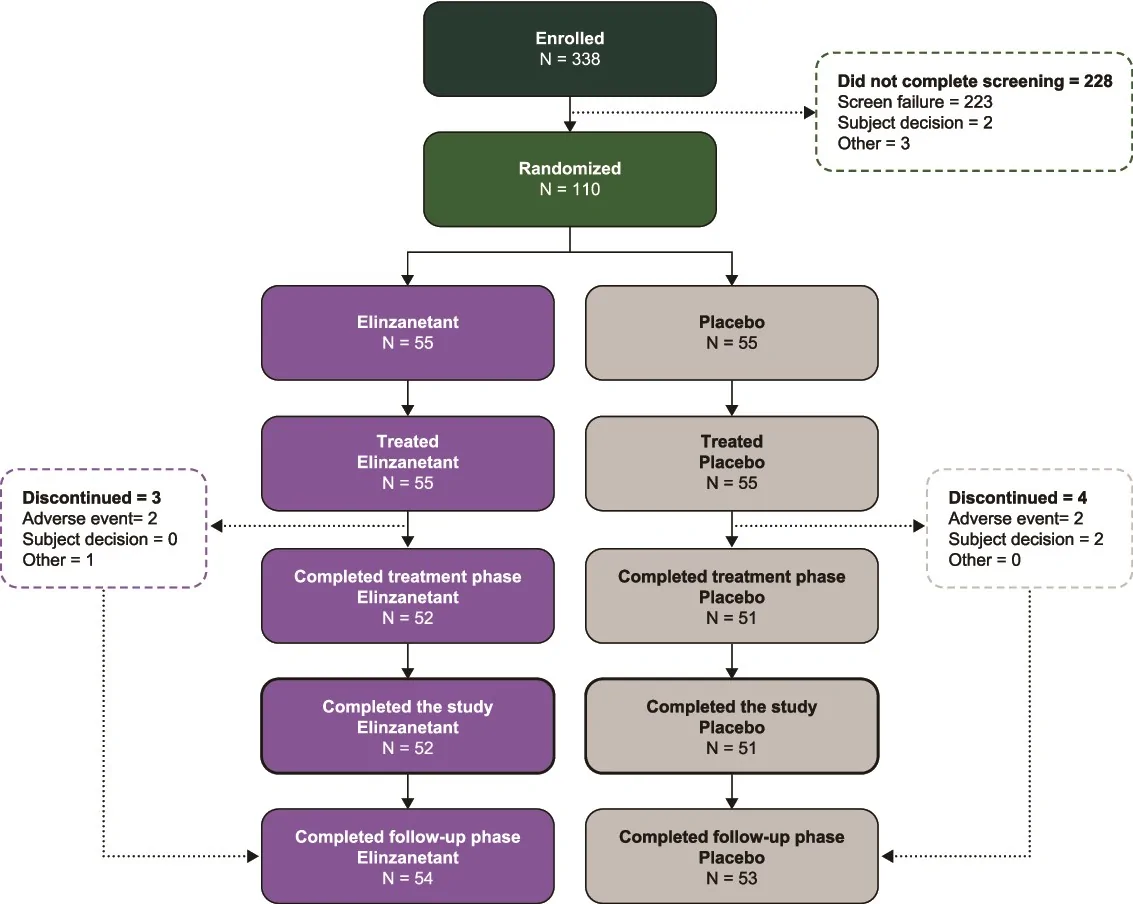
Flow diagram showing participant disposition in the full analysis set FAS, full analysis set (all participants randomly assigned to study treatment, with participants analyzed as per their randomized study treatment according to intention to treat principles).

Treatment arms were generally well balanced regarding demographic and other baseline characteristics, summarized in Table 1. Most participants in the FAS were White (77.3%) and college-educated (60.0%), with no smoking history (74.5%), light alcohol consumption (64.5%), and moderate caffeine consumption (mean, 131.4 mg/day). Mean age and BMI were 54.8 years and 27.8 kg/m^2^, respectively. More than half (56.4%) of participants experienced ≥50 moderate-to-severe VMS/week at baseline. Medical history findings were balanced across treatment arms, as was concomitant medication use, most typically with analgesics, vitamins, renin-angiotensin agents, and lipid modifying agents. Mood disorders are common in postmenopausal women; while there is potential for mood disorders to influence sleep-related outcomes, the two treatment groups were largely balanced regarding anxiety level at baseline, assessed via General Anxiety Disorder-7 (GAD-7) questionnaire. GAD-7 indicated moderate-to-severe anxiety in only a small number of participants (N = 20) at baseline.

**Table 1:** Demographic and baseline characteristics (FAS)

	Elinzanetant 120 mg N = 55 (100%)	Placebo N = 55 (100%)	Total N = 110 (100%)
**Race**, n (%)			
White	40 (72.7%)	45 (81.8%)	85 (77.3%)
Black or African American	14 (25.5%)	9 (16.4%)	23 (20.9%)
Not reported	1 (1.8%)	1 (1.8%)	2 (1.8%)
**Ethnicity**, n (%)			
Not Hispanic or Latino	36 (65.5%)	35 (63.6%)	71 (64.5%)
Hispanic or Latino	18 (32.7%)	20 (36.4%)	38 (34.5%)
Not reported	1 (1.8%)	0	1 (0.9%)
**Age**, years, mean (SD)	54.8 (4.6)	54.9 (4.7)	54.8 (4.6)
**BMI**, kg/m^2^, mean (SD)	28.0 (4.4)	27.6 (3.8)	27.8 (4.1)
**Average weekly frequency of moderate-to-severe VMS**, n (%)			
<35	13 (23.6%)	11 (20.0%)	24 (21.8%)
≥35–<50	11 (20.0%)	13 (23.6%)	24 (21.8%)
≥50	31 (56.4%)	31 (56.4%)	62 (56.4%)
**Baseline PROMIS SD SF 8b total T-score**, mean (SD)	60.9 (5.4)	59.9 (5.5)	60.4 (5.5)
**Baseline Apnea-Hypopnea Index**, /h, median (Q1, Q3)	2.4 (1.1, 4.8)	1.7 (0.4, 3.9)	NA
**Periodic Limb Movement Arousal Index**, /h, median (Q1, Q3)	0.3 (0.0, 2.4)	1.0 (0.0, 2.5)	NA
**History of oophorectomy**, n (%) (SAF)	14 (25.5%)	19 (34.5%)	33 (30.0%)
**Smoking history**, n (%)			
Never	40 (72.7%)	42 (76.4%)	82 (74.5%)
Former	5 (9.1%)	10 (18.2%)	15 (13.6%)
Current	10 (18.2%)	3 (5.5%)	13 (11.8%)
**Alcohol consumption**, n (%)			
Abstinent	14 (25.5%)	25 (45.5%)	39 (35.5%)
Light	41 (74.5%)	30 (54.5%)	71 (64.5%)
**Caffeine consumption**, mg/day, median (Min, Max)^*^	102.0 (1.4, 658.8)	122.4 (1.1, 306.0)	112.2 (1.1, 658.8)
**Level of education**, n (%)			
College or university education	32 (58.2%)	34 (61.8%)	66 (60.0%)
Professional certification	3 (5.5%)	8 (14.5%)	11 (10.0%)
Attending college	5 (9.1%)	3 (5.5%)	8 (7.3%)
Other	14 (25.5%)	10 (18.2%)	24 (21.8%)
Missing	1 (1.8%)	0	1 (0.9%)

^*^As reported by 47 participants in the elinzanetant group and 43 participants in the placebo group.BMI, body mass index; FAS, full analysis set (all participants randomly assigned to study treatment, with participants analyzed as per their randomized study treatment according to intention to treat principles); N, number of participants; n, number of participants with event; NA, not available; PROMIS SD SF 8b, Patient-Reported Outcomes Measurement Information System Short Form 8b; SAF, safety analysis set; SD, standard deviation; VMS, vasomotor symptoms.

### Treatment effects on PSG measures of sleep continuity and sleep architecture

At baseline, mean (SD) WASO was 74.7 (34.8) minutes in the elinzanetant group and 82.8 (34.4) minutes in the placebo group, well above the threshold (>30 minutes) typically required for inclusion in insomnia trials [52]. At Week 4, mean (SD) WASO was 54.9 (24.9) minutes in the elinzanetant group and 77.4 (41.6) minutes in the placebo group, corresponding to mean (SD) changes from baseline of –19.4 (31.1) minutes with elinzanetant and –5.5 (42.7) minutes with placebo. Pre-specified primary analysis showed that the corresponding LS-means difference between groups was –22.5 (90% CI: –35.47, –9.52) minutes favoring elinzanetant (Figure 2A and Table 2). At Week 4, 15.4% (8/52) of participants in the elinzanetant group had WASO <30 minutes, compared with 7.5% (4/53) in the placebo group.

**Table 2:** Analyses of PSG WASO (FAS)

	Elinzanetant 120 mg N = 55	Placebo N = 55	Elinzanetant 120 mg vs Placebo
**Descriptive analysis:** Value at visit: mean (SD), median (IQR) minutes			
Baseline	74.65 (34.83), 72.00 (42.25); n = 55	82.78 (34.35), 76.75 (48.50); n = 55	NA
Week 4	54.85 (24.91), 52.50 (32.63); n = 52	77.43 (41.59), 68.75 (46.00); n = 53	NA
Week 12	59.55 (37.42), 47.25 (53.25); n = 52	64.06 (26.78), 64.00 (34.00); n = 51	NA
**Descriptive analysis:** Change from baseline: mean (SD), median (IQR) minutes			
Week 4	–19.38 (31.09), –15.25 (36.13); n = 52	–5.51 (42.74), –5.50 (44.75); n = 53	NA
Week 12	–14.69 (40.72), –18.00 (53.50); n = 52	–18.36 (35.65), –15.50 (43.75); n = 51	NA
**Primary estimand:** MMRM, LS-mean change from baseline (SE) minutes^*^			*LS-means difference (90% CI)*
Week 4	–23.50 (4.63); n = 47	–1.01 (4.68); n = 47	–22.50 (–35.47, –9.52)
Week 12	–16.48 (4.64); n = 46	–15.54 (4.76); n = 43	–0.94 (–13.96, 12.09)
**Supplementary estimand:** MMRM, LSGM ratio to baseline (SE)^*,†^			*LSGM treatment ratio (90% CI)*
Week 4	0.68 (1.07); n = 47	0.94 (1.07); n = 47	0.72 (0.59, 0.87)
Week 12	0.67 (1.08); n = 46	0.76 (1.09); n = 43	0.88 (0.70, 1.11)
**Post hoc analysis:** Random coefficient model^‡^			
LS-mean change from baseline (SE) minutes	–19.75 (3.03); n = 53	–9.67 (3.00); n = 53	*LS-means difference (95% CI):* –10.07 (–18.54, –1.60)
LSGM ratio to baseline (SE)^‡^	0.69 (1.05); n = 53	0.84 (1.05); n = 53	*LSGM treatment ratio (95% CI):* 0.82 (0.70, 0.95)
Relative change (%) from baseline (95% CI)	–31.47 (–38.27, –23.92); n = 53	–16.10 (–24.38, –6.91); n = 53	*Relative change (%) (95% CI):* –18.32 (–29.56, –5.29)

^*^The mean value of the two consecutive nights at each visit was calculated. If a participant only had one PSG night for a given visit, the data from that one night were used for the analysis. ^†^Analysis used log-transformed data. ^‡^Captures change from baseline over the 12-week treatment period.CI, confidence interval; FAS, full analysis set (all participants randomly assigned to study treatment, with participants analyzed as per their randomized study treatment according to intention to treat principles); IQR, interquartile range; LS, least squares; LSGM, least squares geometric mean; MMRM, mixed model repeated measures; N, number of participants; n, number of participants with observed data and considered in the analysis; NA, not applicable; PSG, polysomnography; SD, standard deviation; SE, standard error; WASO, wakefulness after sleep onset.

**Figure 2:**
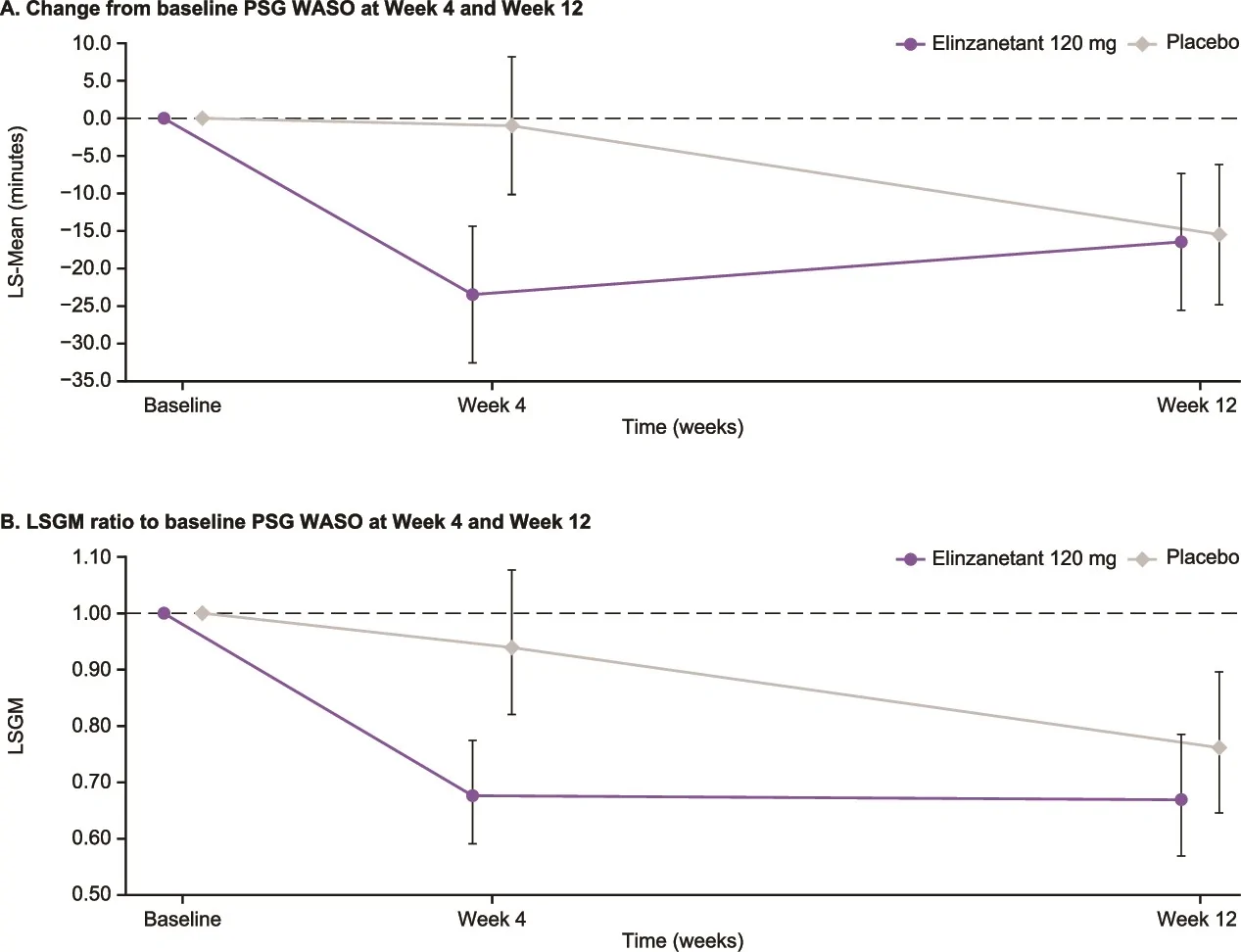
Primary endpoint change from baseline in PSG WASO (in minutes) at Week 4 and secondary endpoint change from baseline in PSG WASO (in minutes) at Week 12 (FAS) (A) primary estimand: Points show LS-mean change from baseline in PSG WASO at Week 4 (primary endpoint) and Week 12 (secondary endpoint), error bars show 90% CI. Dashed horizontal line at 0.0 is a reference line for no change from baseline. (B) Supplementary estimand: Points show LSGM ratio to baseline PSG WASO at Week 4 (primary endpoint) and Week 12 (secondary endpoint), error bars show 90% CI. Dashed horizontal line at 1.00 is a reference line for no change from baseline. CI, confidence interval; FAS, full analysis set (all participants randomly assigned to study treatment, with participants analyzed as per their randomized study treatment according to intention to treat principles); LS-mean; least square mean; LSGM; least square geometric mean; PSG, polysomnography; WASO, wakefulness after sleep onset. Data also presented in Table 2.

At Week 12, mean (SD) WASO was 59.6 (37.4) minutes in the elinzanetant group and 64.1 (26.8) minutes in the placebo group, corresponding to mean (SD) changes from baseline of –14.7 (40.7) minutes with elinzanetant and –18.4 (35.7) minutes with placebo. The corresponding pre-specified primary analysis LS-means difference between groups was –0.9 (90% CI: –14.0, 12.1) minutes, indicating minimal difference between treatment arms (Figure 2A). The lower treatment difference compared to placebo at Week 12 was mainly driven by the higher WASO improvement in the placebo group at Week 12 compared to Week 4. Despite this relative improvement in the placebo arm, the proportion of participants with WASO <30 minutes was greater and remained so in the elinzanetant group (25.0%, 13/52) than in the placebo group (9.8%, 5/51) at Week 12.

The pre-specified supplementary estimand using log-transformed WASO data provided further estimation of treatment effect at the two time points; residual analysis after fitting the MMRM for the primary estimand indicated deviation from the assumption of normality, which can bias the results. The LSGM ratio to baseline (SE) for elinzanetant 120 mg is effectively the same at both Week 4 (0.68 [1.07]) and Week 12 (0.67 [1.08]), showing that the treatment group aggregated improvement in WASO is maintained during the treatment period for elinzanetant. In contrast, the LSGM ratio to baseline for placebo at Week 12 (0.76 [1.09]) is lower compared to Week 4 (0.94 [1.07]). The LSGM ratio (90% CI) between treatments is 0.72 (0.59, 0.87]) and 0.88 (0.70, 1.11) at Week 12, showing a reduction in WASO of 28% at Week 4 and 12% at Week 12 when comparing elinzanetant with placebo (Figure 2B).

Results of the exploratory post hoc analysis of PSG WASO across the entire 12-week period provided additional evidence favoring elinzanetant compared to placebo, with an LS-means difference (95% CI) in change from baseline over the 12-week treatment period of –10.1 (–18.54, –1.60) minutes per night, as shown in Figure 3A. The LSGM treatment ratio (elinzanetant vs placebo), computed using log-transformed values, was 0.8 (95% CI: 0.70, 0.95), corresponding to a relative change with elinzanetant compared to placebo of –18.3% (95% CI: –29.56, –5.29) over the 12-week treatment period. Results of all PSG WASO analyses are summarized in Table 2; primary and supplementary estimand for these endpoints are presented in Figure 2; WASO values by treatment and night are shown in Figure 1.

**Figure 3:**
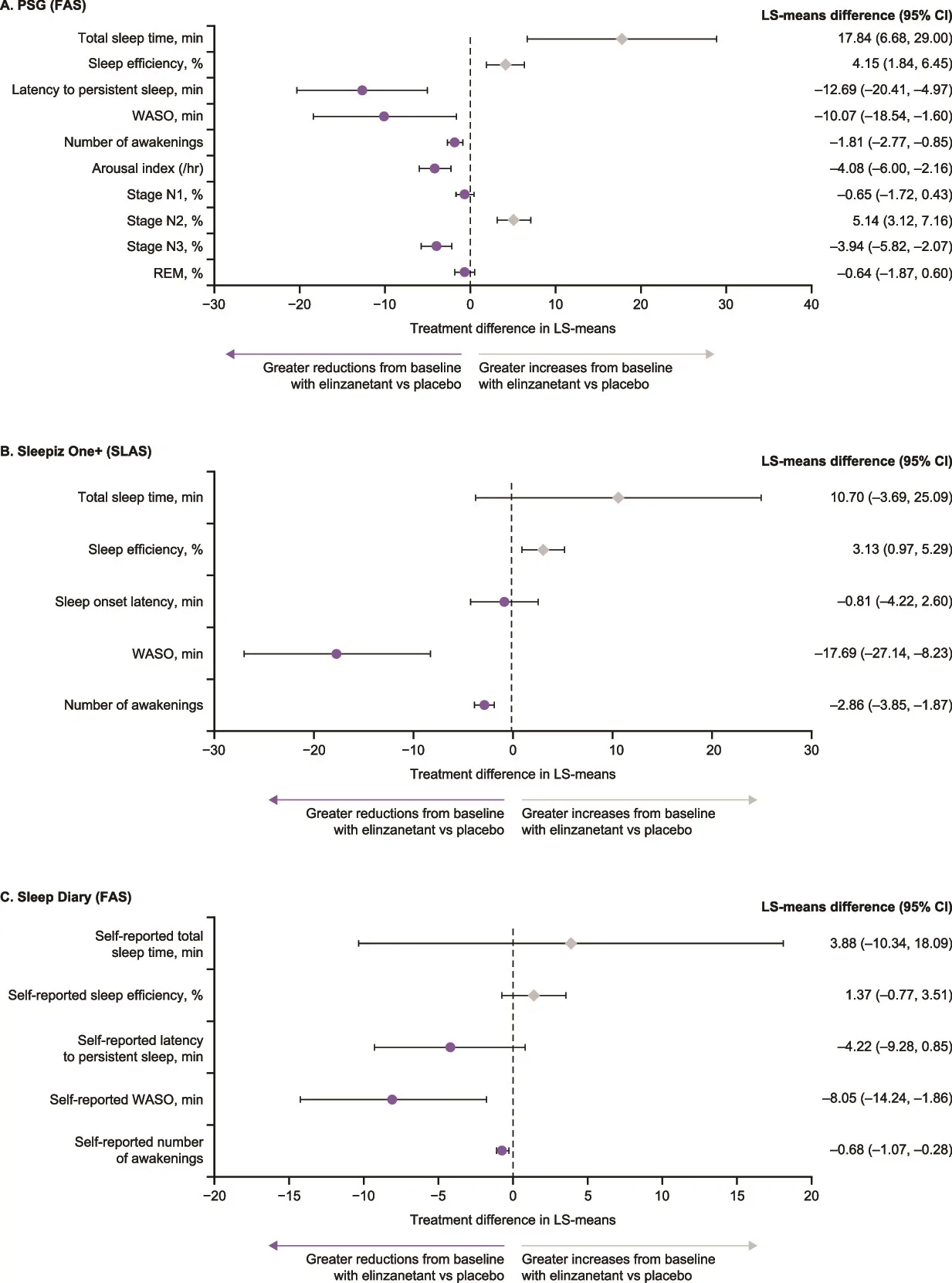
Post hoc analyses showing treatment differences in LS-means for PSG, Sleepiz One+, and Sleep Diary parameters from the random coefficient models across the 12-week period CI, confidence interval; FAS, full analysis set (all participants randomly assigned to study treatment, with participants analyzed as per their randomized study treatment according to intention to treat principles); LS, least squares; PSG, polysomnography; REM, rapid eye movement; SLAS, Sleepiz One+ analysis set; WASO, wakefulness after sleep onset.

Holistic analysis of the remaining PSG parameters, performed using a post hoc random coefficient model, revealed treatment effects (LS-means difference [95% CI]) over the 12-week treatment period on a majority of sleep parameters assessed. Namely, numerically greater increases from baseline in total sleep time (17.8 min [6.68, 29.00]) and sleep efficiency (4.2% [1.84, 6.45]) were observed with elinzanetant compared to placebo, as were greater decreases from baseline in latency to persistent sleep (–12.7 min [–20.41, –4.97]), number of awakenings (–1.8 [–2.77, –0.85]), and arousal index (–4.1/hour [–6.00, –2.16]). Competing treatment effects (LS-means difference [95% CI]) on proportion of time spent in Stage N2 (5.1% [3.12, 7.16]) and Stage N3 (–3.9% [–5.82, –2.07]) sleep were detected; whereas a numerically greater increase from baseline in proportion of time spent in Stage N2 sleep was observed with elinzanetant compared to placebo, an increase from baseline in proportion of time spent in Stage N3 sleep was observed in the placebo group but not in the elinzanetant group. Similar treatment effects on proportion of time spent in Stage N1 (–0.7% [–1.72, 0.43]) and REM (–0.6% [–1.87, 0.60]) sleep were observed. Results from the post hoc analysis are summarized in Table 3 and depicted in Figure 3A. Analyses of Stage N1, N2, N3 and REM sleep duration, along with % sleep, are reported in Table 3.

### Treatment effects on objective sleep parameters assessed nightly with Sleepiz One+

The SLAS included 84 (42 elinzanetant, 42 placebo) of 110 participants in the FAS. Of the remaining 26 participants, 21 did not consent to use the Sleepiz One+ device, and no data were collected with the device for the remaining five. When analyzed across the 12-week treatment period, treatment effects (LS-means difference [95% CI]) favoring elinzanetant were observed on WASO (–17.7 min [–27.14, –8.23]) and number of awakenings (–2.9 [–3.85, –1.87]), with greater decreases from baseline with elinzanetant compared to placebo, and on sleep efficiency (3.1% [0.97, 5.29]), with greater increases from baseline with elinzanetant compared to placebo. In contrast, similar treatment effects were observed between groups on total sleep time (10.7 min [–3.69, 25.09]) and sleep onset latency (–0.8 min [–4.22, 2.60]). Results from the post hoc analysis are summarized in Table 4 and depicted in Figure 3B; change from baseline in WASO over the treatment period is shown in Figure 4A.

**Figure 4:**
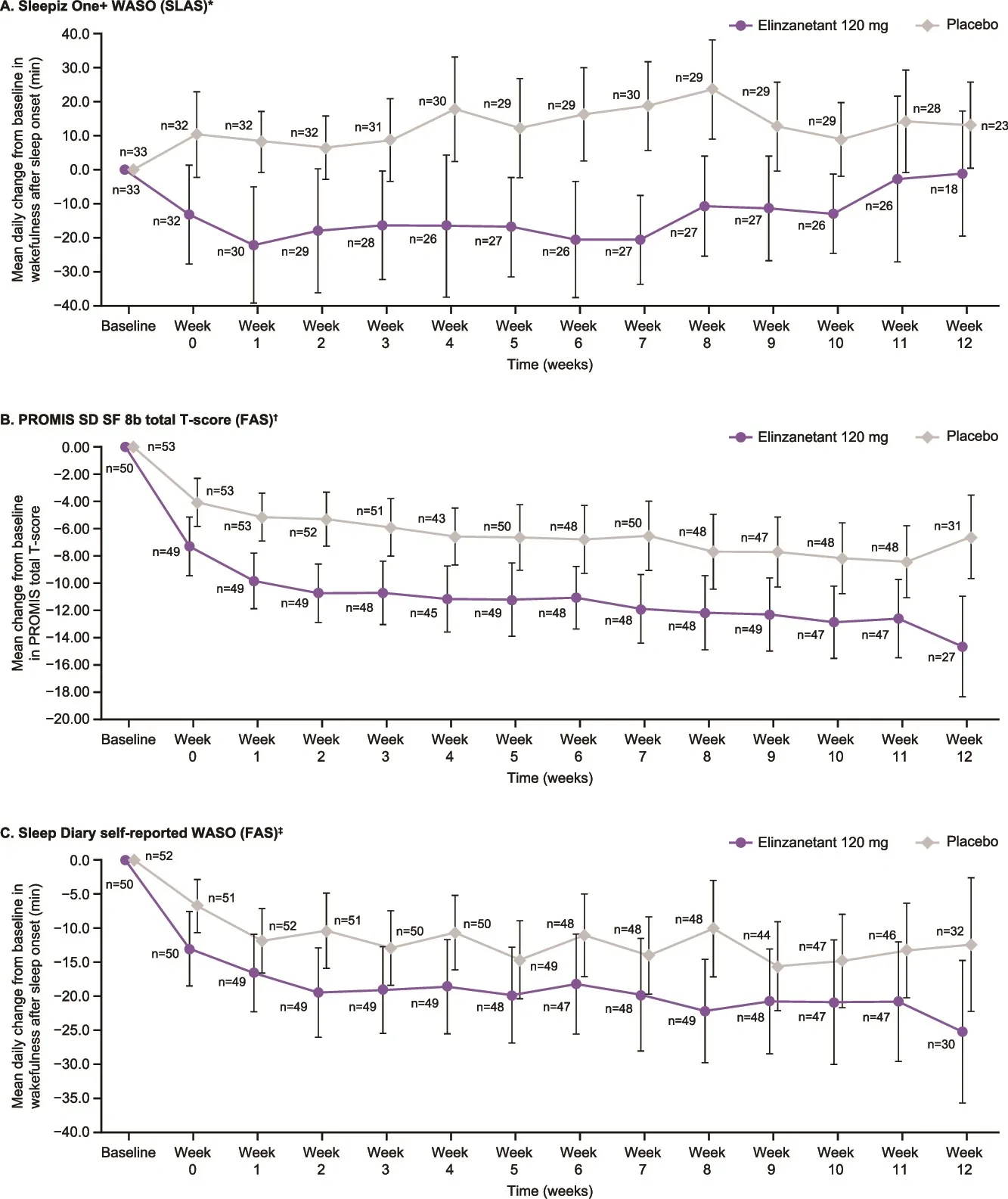
Secondary and exploratory endpoints mean change from baseline in Sleepiz One+ WASO, PROMIS SD -SF 8b total T-scores, and Sleep Diary self-reported WASO each figure displays means with error bars representing 95% CIs. ^*^Mean daily value was derived using all available data assessed at home with a GSD of 4 or more hours for that week. Baseline was derived using all available data assessed at home with a GSD of 4 or more hours prior to starting of treatment. ^†^Participants’ assessment during Weeks 4 and 12 included in-lab PSG nights. ^‡^Mean daily value was derived if a value is provided for at least 4 days within a week, otherwise the value was set to missing for that week. Data collected during Weeks 4 and 12 included in-lab PSG nights. CI, confidence interval; FAS, full analysis set (all participants randomly assigned to study treatment, with participants analyzed as per their randomized study treatment according to intention to treat principles); GSD, good signal duration; PROMIS SD SF 8b, Patient-Reported Outcomes Measurement Information System Short Form 8b; PSG, polysomnography; SLAS, Sleepiz One+ analysis set; WASO, wakefulness after sleep onset.

### Treatment effects on PROs

#### PROMIS SD SF 8b Total T score

At baseline, mean (SD) PROMIS SD SF 8b total T scores were similar in elinzanetant (60.9 [5.4; n = 50]) and placebo (59.9 [5.5; n = 53]) groups, indicating borderline mild/moderate sleep disturbance per established cut-off points [51]. The mean (SD) change from baseline was numerically greater, indicating less sleep disturbance, with elinzanetant compared to placebo throughout the treatment period (Figure 4B), including at Week 4 (elinzanetant, –11.2 [8.0; n = 45]; placebo, –6.6 [6.8; n = 43]) and Week 12 (elinzanetant, –14.7 [9.4; n = 27]; placebo, –6.7 [8.3; n = 31]). Moreover, mean (SD) PROMIS SD SF 8b total T scores of 45.8 (8.7) indicated normalization of sleep disturbance, per established cut-off points, in the elinzanetant group at Week 12 [51]. When considering the averaged effect in an exploratory post hoc analysis using a random coefficient model, elinzanetant demonstrated on average a nearly 4-point greater change from baseline compared to placebo (LS-means difference [95% CI], –3.9 [–6.14, –1.73]).

#### Sleep diary

When analyzed across the 12-week treatment period, treatment effects (LS-means difference [95% CI]) favoring elinzanetant were observed on self-reported WASO (–8.1 min [–14.24, –1.86]) and self-reported number of awakenings (–0.7 [–1.07, –0.28]), with greater decreases from baseline in the elinzanetant group compared to the placebo group. Similar treatment effects were observed on self-reported total sleep time (3.9 min [–10.34, 18.09]), self-reported sleep efficiency (1.4% [–0.77, 3.51]), and self-reported latency to persistent sleep (–4.2 min [–9.28, 0.85]). Results from the post hoc analysis are summarized in Table 5 and depicted in Figure 3C; change from baseline in self-reported WASO over the treatment period is shown in Figure 4C.

### Safety

TEAEs are summarized in Table 3. Overall, the proportion of participants with TEAEs was comparable between groups (elinzanetant, 43.6%; placebo, 49.1%), and almost all were mild or moderate in intensity. Headache (elinzanetant, 9.1%; placebo, 10.9%) was the most frequently reported TEAE in both groups, followed by fatigue (7.3%) in the elinzanetant group and gastroenteritis (5.5%) in the placebo group. Of the most frequently reported TEAEs (≥2 participants/group), fatigue, somnolence, urinary tract infection, and back pain were more common (difference between groups ≥2 participants) in the elinzanetant group while gastroenteritis, increased ALT, increased gamma-glutamyltransferase, and hot flushes were more common in the placebo group. Two (3.6%) participants in the placebo group experienced serious TEAEs, both deemed unrelated to study intervention; no deaths were reported during the study.

**Table 3:** Summary of TEAEs (SAF)

	Elinzanetant 120 mg N = 55 (100%)	Placebo N = 55 (100%)
**Number (%) of participants with TEAEs**		
Any TEAE	24 (43.6%)	27 (49.1%)
Maximum intensity for any TEAE		
Mild	12 (21.8%)	13 (23.6%)
Moderate	12 (21.8%)	13 (23.6%)
Severe	0	1 (1.8%)
Any intervention-related^*^ TEAE	4 (7.3%)	8 (14.5%)
Intervention-related TEAE leading to interruption of intervention	1 (1.8%)	0
Any TEAE related to procedures required by the protocol	0	0
Any TEAE leading to discontinuation of intervention	2 (3.6%)	2 (3.6%)
Any TEAE leading to interruption of intervention	2 (3.6%)	1 (1.8%)
Any TEAE of special interest^†^	1 (1.8%)	4 (7.3%)
Any serious TEAE	0	2 (3.6%)
Any intervention-related^*^ serious TEAE	0	0
Any serious TEAE related to procedures required by the protocol	0	0
Any serious TEAE leading to discontinuation of intervention	0	0
Any serious TEAE leading to interruption of intervention	0	1 (1.8%)

^*^Refers to events with reasonable causal relationship to elinzanetant or placebo as assessed by the investigator. †Pre-specified as postmenopausal uterine bleeding and potential liver events (i.e. any condition triggering close liver observation).

N, number of participants; TEAE, treatment-emergent adverse event; SAF, safety analysis set.

One (1.8%) participant in the elinzanetant group and four (7.3%) participants in the placebo group experienced TEAEs of special interest, all potential liver events. One participant in each group underwent close liver observation. Both cases met liver injury criteria, as assessed by the Liver Safety Monitoring Board; causality to study intervention was assessed as “probable” in the elinzanetant case and “unlikely” in the placebo case. No postmenopausal bleeding was reported in the study.

For clinical laboratory data, no substantial changes over time or differences between treatment groups were observed, and no relevant trends based on descriptive summary statistics were identified.

## Discussion

Elinzanetant improved sleep disturbance associated with menopause, as estimated by a reduction from baseline in PSG WASO at Week 4. Although the estimated change in PSG WASO from baseline at Week 12 was similar to Week 4 in the elinzanetant group, the placebo group showed a larger reduction from baseline at Week 12 than at Week 4. This contributed to an estimated treatment effect of elinzanetant on PSG WASO relative to placebo observed at Week 4 that was less pronounced at Week 12 with overlapping confidence intervals. Reductions in WASO within placebo groups of primary insomnia clinical trials have been previously observed, and this effect was found to accrue over time during the treatment period [53]. High night-to-night variability in WASO data was observed, emphasizing the value of assessing the distinct pattern of menopause-associated sleep disturbance across the 12-week treatment period. Further holistic post hoc random coefficient modeling of PSG, Sleepiz One+, and Sleep Diary WASO data across the entire 12-week period provided support favoring elinzanetant compared to placebo. Nevertheless, more evidence is needed to support the long-term effect of elinzanetant on sleep continuity.

Furthermore, these findings suggest the sleep effects were specific and limited to sleep onset and awakenings during the night, which may be interpreted as a targeted effect on sleep parameters. Additional post hoc analyses on the remaining PSG parameters using the random coefficient model revealed pronounced effects of elinzanetant on the number of nighttime awakenings; participants in the elinzanetant treatment arm experienced, on average, almost three fewer awakenings per night compared to baseline over the entire treatment period. Arousal index was also substantially reduced (>2 fewer arousals per hour in the elinzanetant treatment arm), providing further support for a positive effect of elinzanetant on frequency of arousal from objective sleep measurement. This effect has potential clinical relevance given that frequent nocturnal awakenings are the most common menopausal sleep disturbance, associated with functional impairment and reduced quality of life [5–7].

The reduction in frequency and duration of wakefulness with elinzanetant could be partially attributed to its effect in reducing VMS as shown in the OASIS trials [28–30]. Fezolinetant is a selective NK-3 receptor antagonist, and its effect on VMS is mediated via NK-3 inhibition; sleep effects in the SKYLIGHT trials are likely secondary to VMS reduction indirectly improving sleep [54]. Women with frequent and bothersome VMS are more likely than those without to report frequent nighttime awakenings [55], and nocturnal VMS, as measured with sternal skin conductance, have been directly linked with PSG awakenings and WASO [21, 22, 56]. However, VMS only contribute to a portion of sleep disturbances [21, 22, 56]. Elinzanetant targets KNDy neurons in the hypothalamus via dual NK-1 and NK-3 antagonism. In addition to reducing VMS, elinzanetant may also have a direct effect on sleep. The observed impact of elinzanetant on menopause-associated sleep disturbance suggests a potential role for NK-1 receptor antagonism in sleep regulation. The clinical data of elinzanetant could indicate a similar mechanism in sleep disturbance associated with menopause as in primary insomnia.

Reductions in PSG-derived number of awakenings, arousal index, and WASO were likely key contributors to the positive effects of elinzanetant on PSG-derived total sleep time and sleep efficiency, which increased by 30 minutes and >6%, respectively, in the elinzanetant treatment arm when considered over the 12-week treatment period. PSG-derived latency to persistent sleep was also shorter with elinzanetant, contributing to more time spent asleep. Together, these effects indicate improvements in several PSG indicators of sleep disturbance with elinzanetant in postmenopausal women with moderate-to-severe VMS and WASO above the threshold (>30 minutes) typically required for inclusion in insomnia trials [52]. The increase in PSG-derived total sleep time with elinzanetant was possibly due to an observed increase in Stage N2 sleep duration, though this is speculative and the overall impact on sleep architecture requires further investigation [57]. The change from baseline in Stage N3 percentage across treatment differed between the groups, mostly due to an increase in N3 sleep in the placebo group not seen in the elinzanetant group. There were no significant group differences in Stage N1 or REM sleep with treatment.

PSG provides comprehensive, high-quality sleep data and is unparalleled as an objective assessment tool through its direct measurement of physiological data [41]. However, PSG has inherent limitations, including the unfamiliar sleep environment (in a lab), setup (e.g. use of electrodes, belts and sensors), and narrow observation window [42, 43]. High night-to-night variability in WASO in individuals with sleep disturbance is common, including in women with menopausal insomnia symptoms [13], which PSG data collected over one or two nights cannot capture. In addition, a first night (sleeping worse) and second night (sleeping better) effect has been previously described [58]. To address these limitations, NIRVANA integrated both objective and subjective measures of sleep disturbance on multiple nights in a home sleep environment, including nightly monitoring with the contactless Sleepiz One+ device, which provides a surrogate measure of sleep, and a daily Sleep Diary. Treatment effects favoring elinzanetant were observed for WASO and number of awakenings with both Sleepiz One+ and the Sleep Diary, similar to findings from PSG. For sleep efficiency, treatment effects favoring elinzanetant were observed with PSG and Sleepiz One+, while a numerical trend in the direction of improvement could be observed, though less pronounced, with the Sleep Diary. Treatment effects on total sleep time and latency to persistent sleep (or sleep onset latency with Sleepiz One+), which favored elinzanetant when assessed with PSG, were not replicated with Sleepiz One+ or the Sleep Diary. Treatment effects on PROMIS SD SF 8b total T scores were in line with those previously reported across the OASIS trials [28–30].

Given the notable differences in method and frequency of data collection with PSG (in lab with lights-off to lights-on restricted sleep time; 4 post-baseline nights), Sleepiz One+ (contactless and at home; ≥85 post-treatment nights), and the Sleep Diary, it is unsurprising that there is not perfect concordance for all measures, although results are directionally consistent. Lack of consistency between objective and subjective measures of sleep disturbance is well documented; inclusion of both types of assessments, which may capture different aspects of sleep and recommended in sleep disorders research, is a key strength of this study [59, 60]. Due to these nuanced relationships, non-replication of effects across measures and modalities should be interpreted accordingly. Together, results from this study support reduced wakefulness with elinzanetant, and suggest that a more stable sleep pattern may underpin the improvements in self-reported sleep disturbance observed here and in the OASIS trials [28–30]. Consistent with this observation, number of awakenings (self-reported, actigraphy-based) was the strongest predictor of perceived sleep quality in 50 healthy sleepers completing two consecutive weeks of home monitoring [61]. Likewise, subjective number of awakenings, coupled with subjective total sleep time and patient age, predicted subjective sleep quality with high accuracy in a pooled analysis of insomnia patients [62].

Further work is needed to address the limitations of NIRVANA; namely, its narrow patient population of postmenopausal women experiencing ≥20 moderate-to-severe VMS per week plus sleep disturbance. Exclusion criteria eliminated the most commonly diagnosed sleep disorders; a wide spectrum of sleep disorders are encountered in menopausal women, including those related to increasing rates of sleep-related breathing and movement disorders in postmenopause, which were not the focus of the present work, nor was insomnia disorder [63, 64]. Similarly, as this study focused on menopause-associated sleep disturbance, common comorbidities and contributing factors that occur concurrently in postmenopausal women are not fully reflected in the generally healthy NIRVANA study population. To increase generalizability of results, future studies should include a broader range of midlife women (e.g. perimenopausal women) experiencing menopause-associated sleep disturbance with and without moderate-to-severe VMS. Additional sleep parameters should also be considered, such as wake bouts and analyses of hyperarousal (e.g. high beta EEG activity) [9, 65], which can provide more nuanced insights into menopause-associated sleep disturbance and elinzanetant treatment effects. Expanded characterization of number of awakenings, which were consistently reduced with elinzanetant across assessment modalities, is of particular interest, and future work could evaluate both objective nocturnal VMS and awakenings to investigate pathways for elinzanetant’s effect. Likewise, consideration should be given to understanding the complex relationships between objective measures and subjective perceptions of sleep. Lastly, a full evaluation of the consistencies and differences between PSG and home sleep monitor readings in menopausal women is ongoing. Future publications are planned to address these topics in sufficient detail, as well as expand reporting of PRO-focused secondary and exploratory endpoint results not presented here, which are beyond the scope of this publication.

Elinzanetant is an approved treatment for moderate-to-severe VMS associated with menopause [66, 67], and it has shown consistent and clinically relevant improvements in sleep disturbance, along with a favorable safety profile [28–30]. The percentage of participants with TEAEs in the NIRVANA trial was comparable between treatment groups, and elinzanetant was well-tolerated with safety results in line with previous studies [28–30]. Positive treatment effects on several objective and subjective measures of sleep disturbance were observed in this novel, multimodal, sleep-focused exploratory NIRVANA trial, the first study to integrate gold-standard PSG, continuous home sleep monitoring, and PROs in postmenopausal women with VMS and sleep disturbance. Greater improvements in the primary endpoint of PSG WASO at Week 4 were estimated for the elinzanetant group compared to placebo; however, a greater reduction in PSG WASO in comparison to placebo was not observed at Week 12. Additional studies should include a broader patient population in order to further evaluate the positive effects on sleep continuity and sleep architecture observed in NIRVANA. On a larger scale, continued emphasis should be placed on addressing sleep as a primary driver of poor health-related quality of life for women during the menopausal transition. Studies such as NIRVANA, which can inform menopause management, are critical to improving outcomes for menopausal women, who have traditionally been overlooked and underserved [68].

## Supplementary Material

Supplementary_material_zsag208

## Data Availability

Result summaries of Bayer’s sponsored clinical trials in drug development phases 1, 2, 3, and 4 are provided in the Bayer Trial Finder application after marketing authorization approval in line with the position of the global pharmaceutical industry associations laid down in the “Joint Position on the Disclosure of Clinical Trial Information via Clinical Trial Registries and Databases”. In addition, results of clinical drug trials will be provided on the publicly funded website http://www.ClinicalTrials.gov and EU Clinical Trials Register in line with the applicable regulations. Bayer commits to sharing upon request from qualified scientific and medical researchers patient-level clinical trial data, study-level clinical trial data, and protocols from clinical trials in patients for medicines and indications approved in the United States and European Union on or after January 01, 2014 as necessary for conducting legitimate research. The trial protocol and statistical analysis plan can be accessed at: https://clinicaltrials.gov/study/NCT06112756?tab=study#study-plan

## Notes

Clinical Trial: A Study to Learn About How Elinzanetant Works and How Safe it is in Women Having Sleep Disturbances Associated With Menopause (NIRVANA), https://clinicaltrials.gov/study/NCT06112756, NCT06112756, date of registration 2023-11-01.
