# Supplementary material for: Elinzanetant in postmenopausal women with sleep disturbance: results from the randomized, phase II NIRVANA study
Source: Sleep. 2026 Jul 28;49(10):zsag208. doi: 10.1093/sleep/zsag208 (PMC13658252; doi:10.1093/sleep/zsag208)
Supplement: Supplementary file 1 [file supplementary_material_zsag208.zip › NIRVANA Graphical Plain Language Summary.pdf]

Elinzanetant in postmenopausal women with sleep disturbance: A plain language summary of the NIRVANA sleep study

Fiona C. Baker[1], Hadine Joffe[2], Claudio N. Soares[3], Pauline M. Maki[4], Malek Bajbouj[5,6], Rossella E. Nappi[7,8], Gary Zammit[9,10], Cecilia Caetano[11], Ulrike Krahn[12], Huda Shalhoub[13], Frank Kramer[12], Hoi-Shen Radcliffe[14], Christian Seitz[13,15]

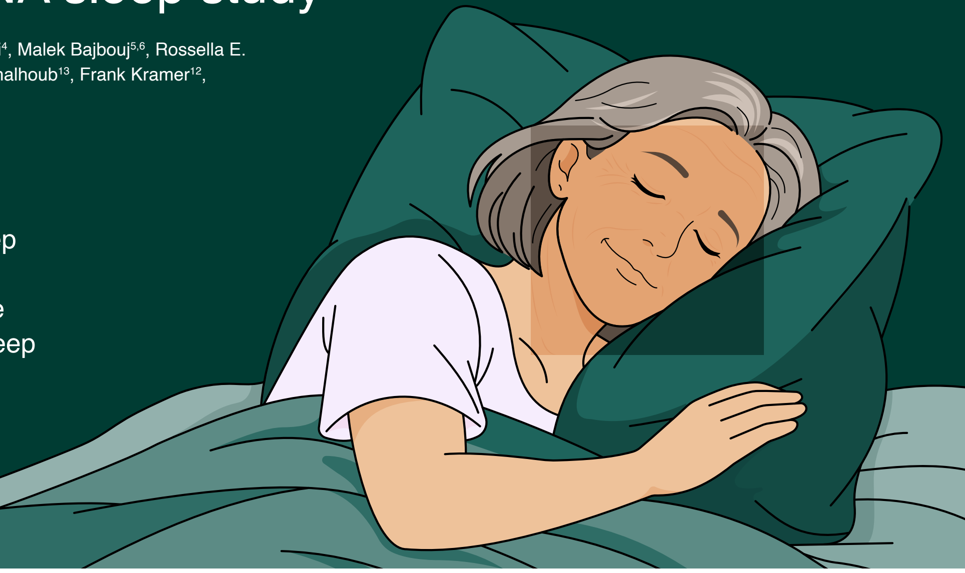

Elinzanetant improved several aspects of sleep in postmenopausal women shown collectively by data from the NIRVANA study, reducing the total time spent awake after initially falling asleep and number of awakenings during the night, and increasing the total time spent asleep.

SLEEP DISTURBANCES IN MENOPAUSE

Sleep disturbances are a common and disruptive symptom of menopause, often meaning broken sleep throughout the night linked to[1-4]:

- Anxiety
- Depression
- Poor quality of life
- Long-term health implications

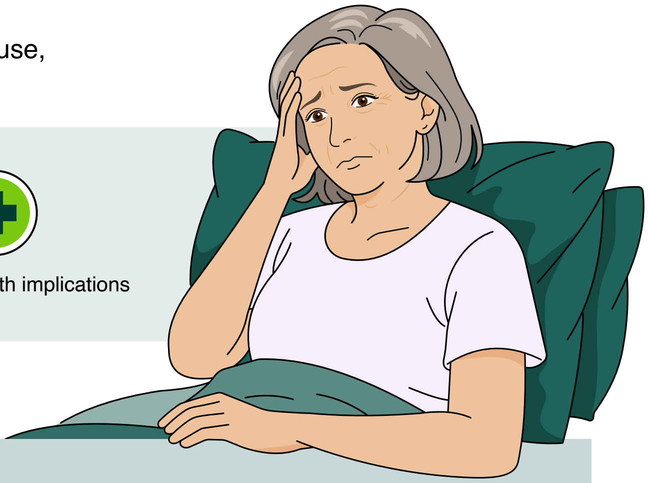

WOMEN EXPERIENCING MENOPAUSE ARE AN UNDERREPRESENTED GROUP IN SLEEP RESEARCH.

WHAT IS ELINZANETANT?

Elinzanetant is a **hormone-free** treatment targeting the source of **hot flashes** in the brain. Elinzanetant blocks two brain signal pathways, **NK-1 and NK-3**, linked to hot flashes and sleep disturbance during menopause.

NK-1 SIGNAL PATHWAY IS THOUGHT TO BE INVOLVED IN SLEEP DISTURBANCES

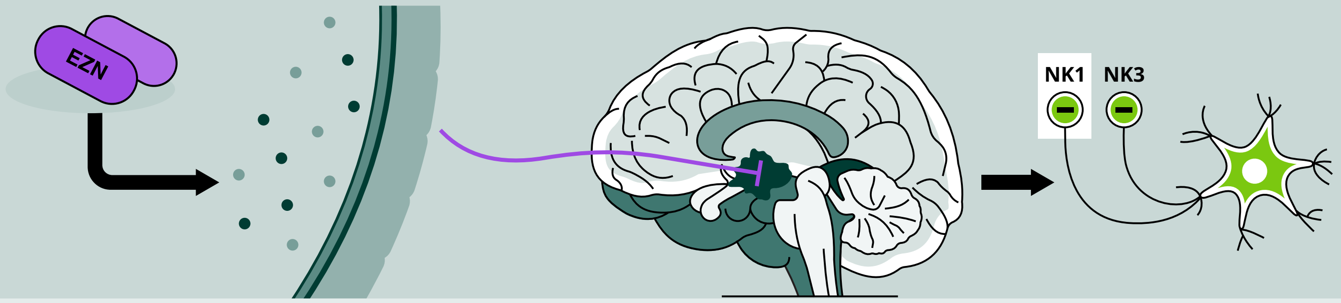

ELINZANETANT AND SLEEP DISTURBANCE IN OTHER STUDIES

Elinzanetant is approved for the treatment of moderate-to-severe hot flashes in women experiencing natural or surgical menopause in multiple countries, and those receiving endocrine therapy for breast cancer in the European Union. Elinzanetant showed improvements in participant-reported sleep disturbance score in Phase III OASIS clinical studies; unlike NIRVANA, women did not have to experience sleep disturbance associated with menopause to enter these studies.

The NIRVANA sleep study was designed to estimate the effect of elinzanetant on sleep disturbance associated with menopause and explore whether the effect of elinzanetant on sleep parameters can be measured using both directly observed and recorded (objective) and participant-reported (subjective) tools and methods.

HOW WAS THE STUDY CARRIED OUT?

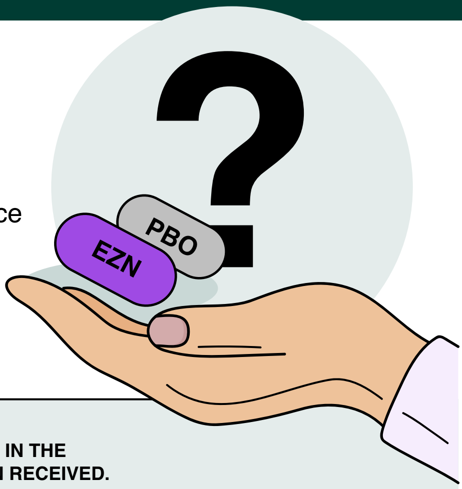

Who took part in the study?

- **110 women** experiencing natural or surgical menopause and sleep disturbance
- Women experiencing **20 or more** moderate-to-severe hot flashes per week
- Women experiencing **30 minutes or more** time spent awake per night after initially falling asleep

110 women

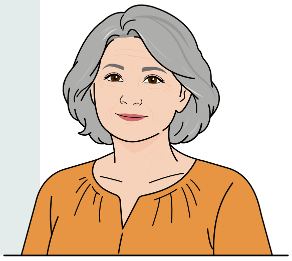

BOTH THE RESEARCHERS AND WOMEN PARTICIPATING IN THE STUDY DID NOT KNOW WHICH TREATMENT THE WOMEN RECEIVED.

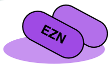

55 women Elinzanetant 120 mg (once daily)

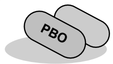

55 women Placebo*

Baseline (before start of treatment) — 1 month — 3 months (end of treatment)

*Inactive substance also referred to as “sugar pill”

HOW WAS MENOPAUSE-ASSOCIATED SLEEP DISTURBANCE MEASURED?

Measured by sleep study and sleep monitoring device

Polysomnography (PSG): Performed within a sleep laboratory using multiple sensors connected to the body to monitor depth and quality of sleep. PSG was performed for 2 consecutive nights at the start of the treatment, and then at 1 month and 3 months for a total of 6 nights.

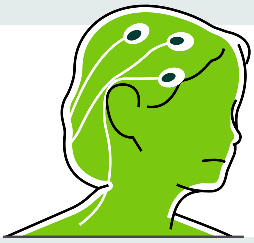

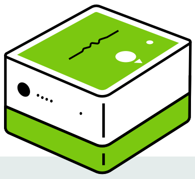

Sleepiz One+: A contactless home-monitoring device placed on a bedside table for nightly sleep monitoring. The device was used every night for the entire treatment period in a subset of women who consented to device use.

Measured by participant-reported outcomes

Sleep disturbance questionnaire: Women filled the questionnaire (weekly) on how well they felt they slept during the treatment period.

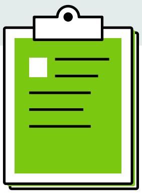

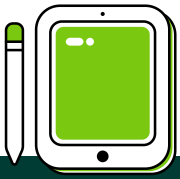

Sleep Diary: Women filled out electronic questionnaire developed by the sponsor which was completed daily in the morning after waking up during the treatment period.

WHAT WERE THE MAIN OBJECTIVES OF THE STUDY?

The main aim was to explore the effect of elinzanetant on sleep disturbance associated with menopause measured by PSG. The secondary aim was to explore this effect of elinzanetant as determined by patient-reported outcomes. Additionally, the study also explored this effect as measured by Sleepiz One+.

Elinzanetant 120 mg (EZN) | Placebo (PBO)

WHAT WERE THE RESULTS?

Women treated with elinzanetant spent less time awake after initially falling asleep than women treated with placebo at 1 month.

At 3 months, women taking placebo also showed improvements, possibly due to changes in sleep habits and data values that were outside the typical range which may have skewed the average.

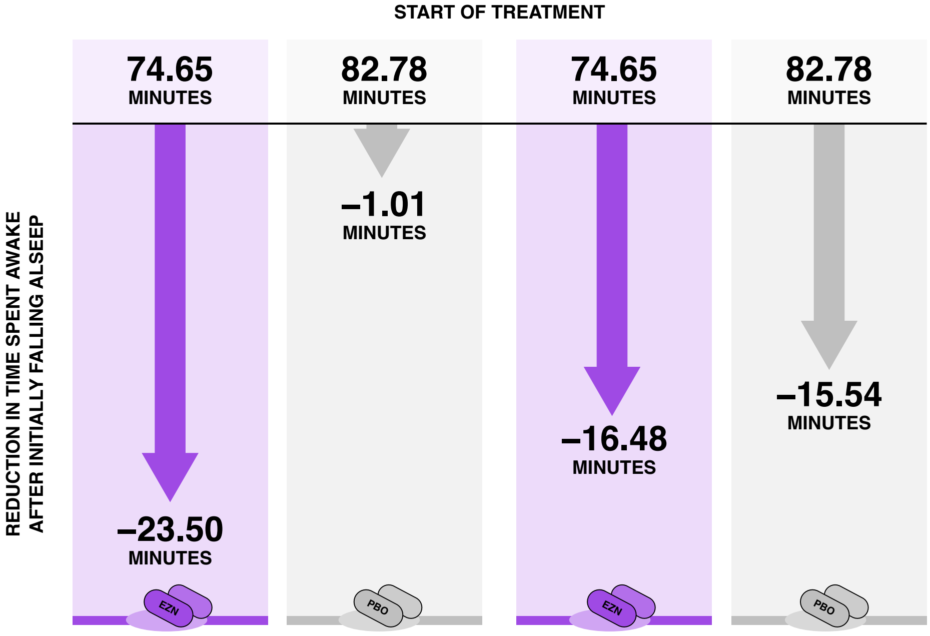

More women had less than 30 minutes of time spent awake after initially falling asleep with elinzanetant than placebo at both 1 month and 3 months.

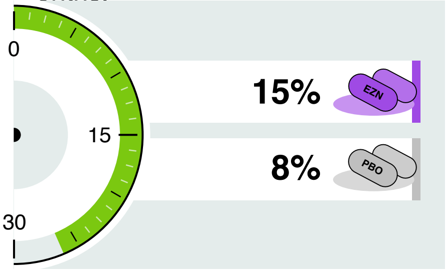

1 MONTH AFTER STARTING TREATMENT

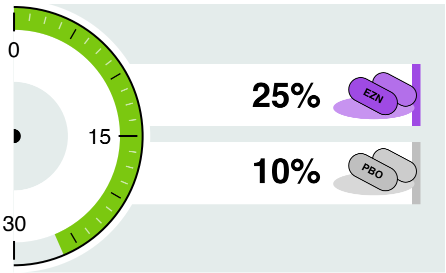

3 MONTHS AFTER STARTING TREATMENT

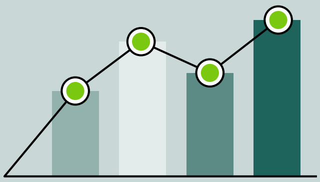

Sleep disturbance during menopause can vary from one night to the next. Therefore, improvements in sleep disturbances were compared between treatment groups averaged over the 3 months when treatment was taken, rather than focusing on individual nights. Using this approach, a difference between elinzanetant and placebo was observed.

ELINZANETANT HAD SIMILAR EFFECTS ON RELEVANT ASPECTS OF SLEEP DISTURBANCE REGARDLESS OF WHICH OF THE THREE METHODS OR TOOLS WERE USED TO MEASURE SLEEP

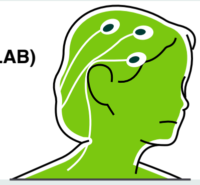

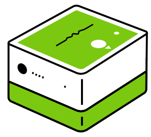

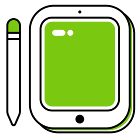

RESULTS WERE ACROSS THE 3-MONTH PERIOD	PSG (IN SLEEP LAB)		SLEEPIZ ONE+ (AT HOME)		SLEEP DIARY (AT HOME)	
	EZN	PBO	EZN	PBO	EZN	PBO
Time spent awake after initially falling asleep (lower value = better sleep)	–19.75 MINUTES	–9.67 MINUTES	–9.74 MINUTES	+7.95 MINUTES	–19.62 MINUTES	–11.57 MINUTES
Number of awakenings (lower value = better sleep)	–2.69	–0.88	–2.05	+0.81	–1.45	–0.78
Total time spent asleep (higher value = better sleep)	+30.09 MINUTES	+12.24 MINUTES	+17.50 MINUTES	+6.79 MINUTES	+39.55 MINUTES	+35.67 MINUTES

Elinzanetant 120 mg | Placebo

WHAT WERE THE RESULTS FROM THE SLEEP DISTURBANCE QUESTIONNAIRE?

Women in both treatment groups experienced mild-to-moderate sleep disturbance associated with menopause at the start of the treatment.

While the total scores for self-reported sleep disturbance reduced in both groups after 1 and 3 months of treatment, the sleep disturbance score became normal compared to an average American adult in the elinzanetant group after 3 months.

The decrease in sleep disturbance score reported by women who took elinzanetant was almost 4x greater compared to women who took the placebo over the 3-month treatment period.

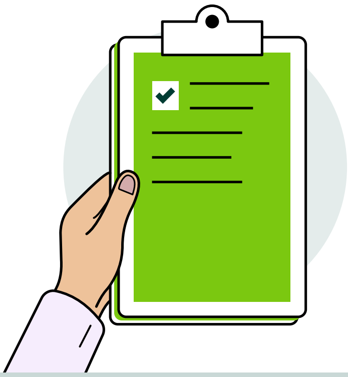

WHAT WERE THE SAFETY RESULTS?

The percentage of side effects reported over 3 months were similar in both treatment groups.
Most common side effects were:

	EZN	PBO
**Headache**	9.1%	10.9%
**Fatigue**	7.3%	0%

NO SERIOUS SIDE EFFECTS WERE REPORTED WITH ELINZANETANT.

WHAT DO THE RESULTS MEAN?

Elinzanetant may reduce the time spent awake after initially falling asleep in postmenopausal women with moderate-to-severe hot flashes and sleep disturbance. Treatment effects of elinzanetant were more pronounced after 1 month of treatment than after 3 months, likely due to placebo effect and outliers. Variability in data between nights was high; effects of elinzanetant compared to placebo were observed when data over all nights were considered. Similar results were indicated across different methods or tools used to measure sleep disturbance.

NIRVANA is the first sleep-focused study in women experiencing sleep disturbance associated with menopause combining multiple methods including:

PSG

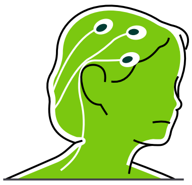

CONTACTLESS HOME SLEEP MONITORING DEVICE

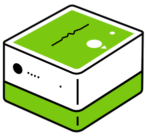

PARTICIPANT-REPORTED SLEEP OUTCOMES

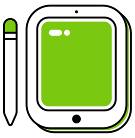

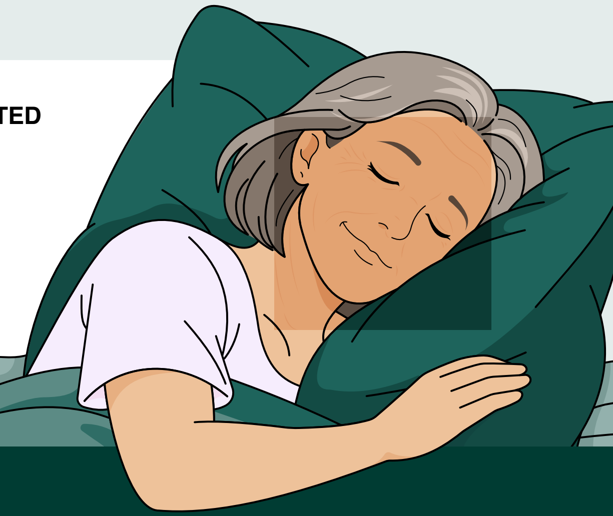

Affiliations:
[1]Center for Health Sciences, SRI International, Menlo Park, CA, USA; [2]Beth Israel Deaconess Medical Center, Harvard Medical School, Boston, MA, USA; [3]Department of Psychiatry, Queen's University, Kingston, Ontario, Canada; [4]University of Illinois Chicago, Chicago, IL, USA; [5]Department of Psychiatry, Charité Universitätsmedizin Berlin, Berlin, Germany; [6]German Center for Mental Health (DZPG), Berlin, Germany; [7]Department of Clinical, Surgical, Diagnostic and Pediatric Sciences, University of Pavia, Pavia, Italy; [8]Research Center for Reproductive Medicine, Gynecological Endocrinology and Menopause, IRCCS San Matteo Hospital, Pavia, Italy; [9]Clinilabs Drug Development Corporation, Eatontown, NJ, USA; [10]Icahn School of Medicine at Mount Sinai, New York, NY, USA; [11]Bayer Consumer Care AG, Basel, Switzerland; [12]Bayer AG, Wuppertal, Germany; [13]Bayer AG, Berlin, Germany; [14]Bayer PLC, Reading, UK; [15]Institute of Clinical Pharmacology and Toxicology, Charité Universitätsmedizin Berlin, Berlin, Germany

References:
1. Baker FC, et al. Nat Sci Sleep. 2018;10:73–95. **2.** Kravitz HM, et al. Sleep. 2008;31(7):979–990. **3.** Attarian H, et al. Menopause. 2015;22(6):674–684. **4.** Kagan R, et al. Menopause. 2021;28(10):1176–1180.

Disclaimer:
This plain language summary represents the opinions of the authors.
For a full list of declarations, including funding and author disclosure statements, and copyright information, please see the full text online.
For more information on elinzanetant, please access the Prescribing Information or Summary of Product Characteristics.
