# Supplementary material for: Elinzanetant in postmenopausal women with sleep disturbance: results from the randomized, phase II NIRVANA study
Source: Sleep. 2026 Jul 28;49(10):zsag208. doi: 10.1093/sleep/zsag208 (PMC13658252; doi:10.1093/sleep/zsag208)
Supplement: Supplementary file 1 [file supplementary_material_zsag208.zip › NIRVANA_MS_Supplementary_Files_Sleepj_zsag208.docx]

**Elinzanetant in postmenopausal women with sleep disturbance: results from the randomized, phase II NIRVANA study**

**Authors:** Fiona C. Baker^1^, Hadine Joffe^2^, Claudio N. Soares^3^, Pauline M. Maki^4^, Malek Bajbouj^5,6^,
Rossella E. Nappi^7,8^, Gary Zammit^9^, Cecilia Caetano^10^, Ulrike Krahn^11^, Huda Shalhoub^12^, Frank Kramer^11^, Hoi-Shen Radcliffe^13^, Christian Seitz^12,14^

**Author Affiliations:**

^1^Center for Health Sciences, SRI International, Menlo Park, CA, USA

^2^Beth Israel Deaconess Medical Center, Harvard Medical School, Boston, MA, USA

^3^Department of Psychiatry, Queen’s University, Kingston, Ontario, Canada

^4^University of Illinois Chicago, Chicago, IL, USA

^5^Department of Psychiatry, Charité Universitätsmedizin Berlin, Berlin, Germany

^6^German Center for Mental Health (DZPG), Berlin, Germany

^7^Department of Clinical, Surgical, Diagnostic and Pediatric Sciences, University of Pavia, Pavia, Italy

^8^Research Center for Reproductive Medicine, Gynecological Endocrinology and Menopause, IRCCS San Matteo Hospital, Pavia, Italy

^9^Clinilabs Drug Development Corporation, Eatontown, New Jersey, USA

^10^Bayer Consumer Care AG, Basel, Switzerland

^11^Bayer AG, Wuppertal, Germany

^12^Bayer AG, Berlin, Germany

^13^Bayer PLC, Reading, UK

^14^Institute of Clinical Pharmacology and Toxicology, Charité Universitätsmedizin Berlin, Berlin, Germany

**Corresponding Author:** Fiona C. Baker; [fiona.baker@sri.com](mailto:fiona.baker@sri.com)

**Supplementary Figure S1.** Boxplot of PSG WASO by night (FAS)

Box: 25^th^ to 75^th^ percentile; horizontal line: median; cross: arithmetic mean; vertical lines extend from the box to a distance of at most 1.5 interquartile ranges and any value more extreme is plotted separately.

FAS, full analysis set (all participants randomly assigned to study treatment, with participants analyzed as per their randomized study treatment according to intention to treat principles); PSG, polysomnography; WASO, wakefulness after sleep onset.

**Supplementary Table S1.** Inclusion and exclusion criteria

**Inclusion Criteria**

Participants are eligible to be included in the study only if all the following criteria apply:

1. Females aged 40 to 65 years, inclusive, at signing of informed consent.
2. Being in the postmenopausal period, defined as:
   a. Serum FSH levels >40 mIU/mL and a serum estradiol concentration of <30 pg/mL at screening, AND hysterectomy performed at least 6 weeks prior to signing of informed consent, OR
   b. Surgical bilateral oophorectomy with or without hysterectomy at least 6 weeks prior to signing of informed consent, OR
   c. At least 12 months of spontaneous amenorrhea prior to signing of informed consent, OR
   d. At least 6 months of spontaneous amenorrhea prior to signing of informed consent with serum FSH levels >40 mIU/mL and a serum estradiol concentration of <30 pg/mL.

Women with an intact uterus need to have an endometrial biopsy with a normal result documented at screening.

3. Experiencing VMS associated with menopause, defined as: Participant has completed HFDD for at least 11 days during the 2 weeks prior to the respective eligibility check, and during this period participant has recorded a weekly average of at least 20 moderate or severe HF (including night-time HF).
4. The participant’s self-reported sleep history includes ongoing sleep disturbances associated with menopause characterized by waking up at night and/or poor quality of sleep.
5. The participant’s self-reported ongoing sleep disturbances cause distress or impairment in social, occupational, or other important areas of functioning.
6. WASO of 30 minutes or more (mean of 2 screening PSGs with neither of the 2 nights <20 min).
7. The participant’s self-reported time in bed is between 6 and 9 hours.
8. The participant’s self-reported bedtime is usually between 21.00 and 24.00 hours and it does not vary by more than ±2 hours.
9. Negative urine pregnancy test at screening in participants with intact uterus and at least one ovary.
10. BMI between 18 and 38 kg/m^2^ at screening.
11. Capable of giving signed informed consent as described in Section 10.1.3, which includes compliance with the requirements and restrictions listed in the ICF and in this protocol.
12. Normal breast cancer screening result available prior to start of treatment in line with the method and frequency recommended in the respective country. The time elapsed since the last screening examination should be sufficiently short that no further routine screening examination needs to be scheduled during the course of the study.
13. In individuals with a cervix: normal cervical cancer screening result available prior to start of treatment in line with the method and frequency recommended in the respective country. The time elapsed since the last screening examination should be sufficiently short that no further routine screening examination needs to be scheduled during the course of the study.

**Exclusion Criteria**

Participants are excluded from the study if any of the following criteria apply:

1. Medical history includes a diagnosis of a sleep disorder other than sleep disturbances associated with the menopause (e.g., sleep apnea, restless leg syndrome, circadian rhythm sleep disorder).
2. Clinically significant abnormalities that interfere with the study goals, in the opinion of the investigator, based on the medical history, physical examination, vital signs, clinical laboratory tests, eC-SSRS, and other assessments completed during screening.
3. Current or history (except complete remission for 5 years or more) of any malignancy (except basal and squamous cell skin tumors).
4. Renal impairment greater than moderate (i.e. estimated glomerular filtration rate <30 mL/min/1.73 m^2^) at screening.
5. Abnormal liver parameters (presence of at least one of the following criteria):
   - AST >2 x ULN
   - ALT >2 x ULN
   - AP >2 x ULN
   - TBL >ULN unless explained by Gilbert’s syndrome
   - INR >ULN unless explained by, e.g., intake of anticoagulants
   - Diagnosis of hepatitis B infection, i.e., Hbs-antigen positive at screening
   - Diagnosis of hepatitis C infection, i.e., hepatitis C antibodies and HCV-RNA positive at screening.
6. Has used and is unwilling to wash-out use of any of the prohibited concomitant medications, as specified in Sections 6.9 and 10.7.
7. Inability to comply with the use of prohibited medications as described in Sections 6.9.1 and 10.7.
8. Current cognitive behavioral therapy for sleep disorders.
9. Concurrent (or within 2 months prior to signing of ICF) participation in a clinical study with an investigational medicinal product (including medical devices) with the exception for participants who completed another elinzanetant study.
10. Shift work within 2 weeks prior to the SCR-2 visit, or planned shift work during the study.
11. Apnea hypopnea index (AHI) >15 on the first PSG screening night.
12. Periodic limb movement arousal index (PLMAI) >15 on the first PSG screening night.
13. Travel across ≥3 time zones within 2 weeks prior to the SCR-2 visit, or planned travel across ≥3 time zones during study.
14. Regular napping i.e. >3 naps per week each with duration of >30 minutes.
15. At screening, a suicidal ideation in the past 6 months (as indicated by “Yes” on item 4 or item 5 of the suicidal ideation section of the eC-SSRS) or suicidal behavior in the past 6 months (indicated by “Yes” on any item of the suicidal behavior section of the eC-SSRS).
16. Clinically relevant drug or alcohol abuse within 12 months of signing of informed consent.
17. Use of substances that in the opinion of the investigator contribute to the participant’s sleep disturbances (e.g. caffeine, alcohol, cannabis and its derivatives).
18. Dependent on the investigator, the contract research organization(s) or Sponsor for education or employment (e.g. family members, employees, people who receive grants/education).
19. Known hypersensitivity to elinzanetant or any of the excipients in the formulation.
20. Inability to comply with the study procedures for any reason, including the following examples: language comprehension, psychiatric illness, general inability to get to the study site or ePRO completion.
21. Current diagnosis of the following pre-malignant conditions: cervical dysplasia; breast atypia including atypical ductal hyperplasia, atypical lobular hyperplasia, lobular carcinoma in situ; cirrhosis of the liver; and nodular hepatic lesions.
22. Any unexplained post-menopausal uterine bleeding.

**Supplementary Table S2.** Sleep parameter definitions

| **PSG** |  |
| --- | --- |
| Total sleep time | Duration (min) of total REM and non-REM sleep during time in bed. |
| Time in bed | 8-hour period between lights off and lights on. |
| Sleep efficiency | The percentage of time spent asleep while in bed;  (Total sleep time / Time in bed) x 100. |
| Latency to persistent sleep | Time (min) from lights off to the first epoch of 20 consecutive epochs of non-wakefulness. |
| Wakefulness after sleep onset (WASO) | Time (min) spent awake from onset of persistent sleep to lights-on. Persistent sleep is defined as 20 consecutive epochs (10 min) of non-wakefulness. |
| Number of awakenings | Total number of awakenings from onset of persistent sleep to lights-on. An awakening is defined as a PSG recording of at least 2 consecutive wake epochs. An awakening must be separated by an epoch of Stage N2 or Stage N3 or Stage R. Separation by Stage N1 does not denote a new awakening.  Terminal awakening is not included in the count. Terminal awakening is the last awakening prior to lights-on and the participant remains awake until lights-on. If participant is asleep at lights-on, there is no terminal awakening. |
| Arousal index | The number of brief awakenings per hour of sleep. The index is based on the number of all arousals scored and counted during sleep time reported per hour.  An arousal is an abrupt shift in EEG frequency, which may include theta, alpha, and/or frequencies greater than 16 Hz but not spindles. The minimum duration of an arousal event is 3 seconds, with at least 10 seconds of stable sleep preceding the change. Scoring arousals during REM must also be accompanied by a concurrent increase in submental EMG lasting at least 1 second. |
| Stage N1 | Sleep Time: Total duration (min) in Stage 1 non-REM sleep from lights-off to lights-on.  % Sleep: (Stage N1 sleep time / Total sleep time) x 100 |
| Stage N2 | Sleep Time: Total duration (min) in Stage 2 non-REM sleep from lights-off to lights-on.  % Sleep: (Stage N2 sleep time / Total sleep time) x 100 |
| Stage N3 | Sleep Time: Total duration (min) in Stage 3 non-REM sleep from lights-off to lights-on.  % Sleep: (Stage N3 sleep time / Total sleep time) x 100 |
| Rapid eye movement (REM) | Sleep Time: Total duration (min) in REM sleep from lights-off to lights-on.  % Sleep: (REM sleep time / Total sleep time) x 100 |
| **Sleepiz One+** |  |
| Total sleep time | Total time (min) during the recording when the participant was asleep. |
| Sleep efficiency | Percentage of time the monitored participant was asleep while in bed, from the participant's last entry into bed before falling asleep to the participant's last exit from the bed. |
| Sleep onset latency | Time (min) from the participant’s last entry into bed before falling asleep to the first sleep event with a duration greater or equal to 10 min. |
| Wakefulness after sleep onset (WASO) | Time (min) the monitored participant was awake between the sleep onset (first sleep period longer than 10 min) and the last awakening. |
| Awakening count | Number of times the monitored participant woke up between the sleep onset (first sleep period longer than 10 min) and the last awakening. |
| **Sleep Diary** |  |
| Self-reported total sleep time | Time (in minutes) between going to bed (Item 1) and waking up in the morning (Item 5) minus the time taken to fall asleep (Item 2) and total duration of awakenings (Item 4) |
| Self-reported time in bed | Time (in hours) between going to bed (Item 1) and getting out of bed (Item 6) |
| Self-reported sleep efficiency | (Self-reported total sleep time [in hours] / Self-reported time in bed) * 100 |
| Self-reported latency to persistent sleep | Item 2: time taken to fall asleep (in minutes) |
| Self-reported wakefulness after sleep onset (WASO) | Item 4: total duration of awakenings (in minutes) |
| Self-reported number of awakenings | Item 3: total number of times woke up in the night |

**Supplementary Table S3.** LS-means for PSG parameters from random coefficient model (FAS)

|  | **Baseline (summary statistics)** | | **Change from baseline (random coefficient model)** | | |
| --- | --- | --- | --- | --- | --- |
|  | **Elinzanetant 120 mg, N=55   *Mean (SD)*** | **Placebo N=55   *Mean (SD)*** | **Elinzanetant 120 mg  N=55, n=53*** ***LS-mean (SE)*** | **Placebo  N=55, n=53*** ***LS-mean (SE)*** | **Elinzanetant 120 mg  vs placebo   *Difference in  LS-means  (95% CI)*** |
| **Total sleep time, min** | 372.96 (50.74) | 357.86 (46.61) | 30.09 (3.98) | 12.24 (3.95) | 17.84 (6.68, 29.00) |
| **Sleep efficiency, %** | 77.31 (10.38) | 74.41 (9.56) | 6.59 (0.82) | 2.44 (0.82) | 4.15 (1.84, 6.45) |
| **Latency to persistent sleep, min** | 40.72 (34.07) | 46.56 (31.27) | –15.82 (2.76) | –3.13 (2.74) | –12.69 (–20.41, –4.97) |
| **WASO, min** | 74.65 (34.83) | 82.78 (34.35) | –19.75 (3.03) | –9.67 (3.00) | –10.07 (–18.54, –1.60) |
| **Number of awakenings** | 10.94 (3.47) | 10.72 (3.68) | –2.69 (0.34) | –0.88 (0.34) | –1.81 (–2.77, –0.85) |
| **Arousal index (/hr)** | 12.89 (6.04) | 13.01 (5.29) | –2.11 (0.69) | 1.97 (0.68) | –4.08 (–6.00, –2.16) |
| **Stage N1, %** | 11.04 (4.98) | 11.72 (5.40) | –2.91 (0.38) | –2.26 (0.38) | –0.65 (–1.72, 0.43) |
| **Stage N2, %** | 56.16 (8.40) | 56.81 (8.24) | 4.83 (0.72) | –0.31 (0.72) | 5.14 (3.12, 7.16) |
| **Stage N3, %** | 13.50 (6.65) | 11.81 (7.01) | –2.32 (0.67) | 1.62 (0.67) | –3.94 (–5.82, –2.07) |
| **REM, %** | 19.30 (5.72) | 19.66 (4.63) | 0.37 (0.44) | 1.01 (0.44) | –0.64 (–1.87, 0.60) |
| **Stage N1, min** | 39.94 (16.83) | 40.29 (16.78) | –7.64 (1.27) | –7.17 (1.26) | –0.47 (–4.02, 3.08) |
| **Stage N2, min** | 208.89 (39.48) | 204.79 (42.78) | 36.20 (3.61) | 5.07 (3.59) | 31.13 (21.03, 41.22) |
| **Stage N3, min** | 50.44 (24.68) | 41.54 (23.66) | –5.75 (2.63) | 9.17 (2.62) | –14.91 (–22.33, –7.50) |
| **REM, min** | 73.70 (26.74) | 71.25 (21.00) | 6.75 (1.99) | 5.95 (1.97) | 0.80 (–4.74, 6.35) |

*Number of participants in the treatment arm (N) and number of participants with observed data (n).
CI, confidence interval; FAS, full analysis set (all participants randomly assigned to study treatment, with participants analyzed as per their randomized study treatment according to intention to treat principles); PSG, polysomnography; REM, rapid eye movement; SD, standard deviation; SE, standard error; WASO, wakefulness after sleep onset.

**Supplementary Table S4.** LS-means for Sleepiz One+ parameters from random coefficient model (SLAS)*

|  | **Baseline (summary statistics)** | | **Change from baseline (random coefficient model)** | | |
| --- | --- | --- | --- | --- | --- |
|  | **Elinzanetant 120 mg, N=42   *Mean (SD)*** | **Placebo N=42   *Mean (SD)*** | **Elinzanetant 120 mg  N=42, n=32**^†^ ***LS-mean (SE)*** | **Placebo  N=42, n=33**^†^ ***LS-mean (SE)*** | **Elinzanetant 120 mg  vs Placebo   *Difference in  LS-means  (95% CI)*** |
| **Total sleep time, min** | 370.43 (62.79) | 375.99 (48.72) | 17.50 (5.18) | 6.79 (4.99) | 10.70 (–3.69, 25.09) |
| **Sleep efficiency, %** | 76.26 (11.72) | 78.14 (7.28) | 2.70 (0.78) | –0.43 (0.75) | 3.13 (0.97, 5.29) |
| **Sleep onset latency, min** | 25.69 (14.22) | 25.25 (11.92) | –1.42 (1.23) | –0.61 (1.18) | –0.81 (–4.22, 2.60) |
| **WASO, min** | 77.73 (52.57) | 61.71 (27.58) | –9.74 (3.39) | 7.95 (3.27) | –17.69 (–27.14, –8.23) |
| **Number of awakenings** | 12.58 (4.48) | 12.14 (4.02) | –2.05 (0.36) | 0.81 (0.34) | –2.86 (–3.85, –1.87) |

*Baseline was derived using all available data assessed at home with a good signal duration (GSD) ≥4 hours prior to starting the treatment. Data measured by Sleepiz One+ at home on nights with GSD ≥4 hours are used in the random coefficient model. ^†^Number of participants in the treatment arm (N) and number of participants with observed data (n).
CI, confidence interval; LS, least squares; SD, standard deviation; SE, standard error; SLAS, Sleepiz One+ analysis set; WASO, wakefulness after sleep onset.

**Supplementary Table S5.** LS-means for Sleep Diary parameters from random coefficient model (FAS)

|  | **Baseline (summary statistics)** | | **Change from baseline (random coefficient model)** | | |
| --- | --- | --- | --- | --- | --- |
|  | **Elinzanetant 120 mg, N=55   *Mean (SD)*** | **Placebo N=55   *Mean (SD)*** | **Elinzanetant 120 mg  N=55, n=50*** ***LS-mean (SE)*** | **Placebo  N=55, n=52*** ***LS-mean (SE)*** | **Elinzanetant 120 mg  vs Placebo   *Difference in  LS-means  (95% CI)*** |
| **Self-reported total sleep time, min** | 396.06 (70.20) | 404.91 (60.99) | 39.55 (5.10) | 35.67 (5.03) | 3.88 (–10.34, 18.09) |
| **Self-reported sleep efficiency, %** | 78.51 (9.25) | 78.48 (7.80) | 6.67 (0.77) | 5.30 (0.76) | 1.37 (–0.77, 3.51) |
| **Self-reported latency to persistent sleep, min** | 34.24 (19.11) | 42.95 (27.26) | –13.21 (1.81) | –9.00 (1.78) | –4.22 (–9.28, 0.85) |
| **Self-reported WASO, min** | 42.41 (25.66) | 45.47 (25.60) | –19.62 (2.22) | –11.57 (2.19) | –8.05 (–14.24, –1.86) |
| **Self-reported number of awakenings** | 3.58 (1.72) | 3.49 (1.86) | –1.45 (0.14) | –0.78 (0.14) | –0.68 (–1.07, –0.28) |

*Number of participants in the treatment arm (N) and number of participants with observed data (n).
CI, confidence interval; FAS, full analysis set (all participants randomly assigned to study treatment, with participants analyzed as per their randomized study treatment according to intention to treat principles); LS, least squares; SD, standard deviation; SE, standard error; WASO, wakefulness after sleep onset.

**Supplementary Video Captions**

| **Time:** | **Captions** |
| --- | --- |
| 00:00:00:00 | We present the results from the Phase II NIRVANA study of elinzanetant in postmenopausal women with sleep disturbance. |
| 00:00:07:16 | Hot flashes and sleep disturbances are among the most common and disruptive symptoms experienced by women during menopause and can negatively impact quality of life. |
| 00:00:19:03 | Elinzanetant is a dual neurokinin-targeted therapy, blocking both NK-1 and NK-3 receptors in the brain and NK-1 peripheral receptors in the skin. It is currently approved in multiple countries for the treatment of moderate-to-severe hot flashes associated with menopause, in addition to those caused by endocrine therapy for breast cancer in the European Union. |
| 00:00:42:24 | In the Phase III OASIS clinical program, elinzanetant also showed improvement in patient-reported sleep disturbance based on a weekly sleep disturbance questionnaire. |
| 00:00:53:16 | This led to the sleep-focused Phase II NIRVANA study to estimate the effect and assess safety of elinzanetant for sleep disturbance associated with menopause. |
| 00:01:03:21 | Sleep disturbance was assessed using both objective and subjective measures. Objective measures included polysomnography performed in a sleep laboratory, and Sleepiz One+, a contactless home-monitoring device for nightly sleep monitoring. Subjective measures included electronic patient-reported outcomes using a sponsor-developed sleep diary and other patient-reported questionnaires not discussed in this video. |
| 00:01:31:21 | NIRVANA enrolled postmenopausal women aged 40 to 65 years experiencing 20 or more moderate-to-severe hot flashes every week and nightly sleep disturbance. Sleep disturbance was defined as 30 or more minutes of time spent awake every night after initially falling asleep and before the final wake-up in the morning, also known as wakefulness after sleep onset, or WASO, measured by polysomnography. |
| 00:01:59:04 | Postmenopausal women were randomized 1:1 to receive elinzanetant 120 mg or placebo for 12 weeks. Sleep disturbance was assessed using in-lab polysomnography for 2 consecutive nights per visit before treatment, at Week 4 and Week 12 for a total of 6 nights, Sleepiz One+ nightly, sleep diary filled daily by participants in the morning, and participant-reported weekly sleep disturbance questionnaire over the entire 12-week treatment period. |
| 00:02:32:03 | The primary aim of the study was to observe change in time spent awake after initially falling asleep at Week 4 from before treatment measured by polysomnography. |
| 00:02:42:23 | Elinzanetant demonstrated a greater reduction in time spent awake after initially falling asleep by 23.5 minutes versus 1.0 minute in the placebo group at Week 4. |
| 00:02:53:21 | Additionally, more participants in the elinzanetant group had less than 30 minutes of time spent awake after initially falling asleep than placebo at Week 4. |
| 00:03:04:09 | At Week 12, the reduction in time spent awake after initially falling asleep was only slightly different between the elinzanetant and placebo groups. Participants in the placebo group improved more than expected, possibly due to changes in sleep habits and a few unusually high or low values in the data affecting group averages. |
| 00:03:25:24 | However, the percentage of participants with less than 30 minutes of time spent awake after initially falling asleep was still greater in the elinzanetant group than the placebo group at Week 12. |
| 00:03:37:25 | Inter-night variability between consecutive nights of polysomnography at each visit was noted in both treatment groups. Therefore, all available timepoints for each overnight polysomnography were considered separately to study improvement in sleep disturbance over the entire 12-week treatment period in a post hoc analysis. |
| 00:03:58:10 | When analyzed over the entire 12-week treatment period, elinzanetant showed greater reduction than placebo in time spent awake after initially falling asleep with polysomnography. |
| 00:04:10:06 | Elinzanetant also showed greater reduction in time spent awake after initially falling asleep with Sleepiz One+ measured nightly, |
| 00:04:18:13 | and reduction in time spent awake after initially falling asleep with Sleep Diary measured daily over the entire 12-week treatment period at home. |
| 00:04:27:18 | Higher total sleep time and percentage of time spent asleep in bed, also called sleep efficiency, were observed with elinzanetant compared with placebo with both polysomnography and Sleepiz One+. |
| 00:04:41:10 | Similarly, greater decreases were observed in number of awakenings with elinzanetant compared with placebo across the three measures, and in time to fall asleep with PSG and Sleepiz One+, over the entire 12-week treatment period. |
| 00:04:58:09 | The percentage of adverse events reported over 12 weeks were similar in both treatment groups. Headache was the most common adverse event in both treatment groups, followed by fatigue in the elinzanetant group, consistent with the common adverse events reported in the Phase III OASIS clinical program. No severe or serious adverse events were reported in the elinzanetant group. |
| 00:05:21:21 | In summary, NIRVANA is the first study to integrate multimodal objective and subjective measures of sleep disturbance in postmenopausal women. |
| 00:05:31:06 | Results from the NIRVANA study suggest that elinzanetant may improve objective and subjective sleep disturbance across measures, including time spent awake after initially falling asleep in postmenopausal women with hot flashes and sleep disturbance. |
